# Supplementary material for: The CGG triplet repeat binding protein 1 counteracts R-loop induced transcription-replication stress
Source: EMBO Rep. 2025 Aug 26;26(19):4691–722. doi: 10.1038/s44319-025-00550-1 (PMC12508481; doi:10.1038/s44319-025-00550-1)
Supplement: Supplementary file 1 — Table EV1 [file 44319_2025_550_MOESM1_ESM.docx]

**Table EV1. Oligonucleotides used in this study**

| **Oligo** | **Sequence** | **Description** |
| --- | --- | --- |
| MOV10_ex1_fw | CGGCTGCGGACCATTTATAA | Primer used for RT-qPCR to determine transcription levels of MOV10 |
| MOV10_ex2_rev | CTGCGGTTGGTGGGTTTTAA | Primer used for RT-qPCR to determine transcription levels of MOV10 |
| MALAT1_ex1_fw | AAAAGCAGACCCAGAGCAGT | Primer used for RT-qPCR to determine transcription levels of MALAT1 |
| MALAT1_ex1_rev | CCTGAAAGTGCTCACAAGGC | Primer used for RT-qPCR to determine transcription levels of MALAT1 |
| TLCD1_ex1_fw | CTCTGTCGCCTGCCCCTA | Primer used for RT-qPCR to determine transcription levels of TLCD1 |
| TLCD1_ex1_fw | CGCCGTCTCAATCTCCACTA | Primer used for RT-qPCR to determine transcription levels of TLCD1 |
| UHRF1_ex1_fw | ACTCGCTGTCCAGGCTGA | Primer used for RT-qPCR to determine transcription levels of UHRF1 |
| UHRF1_ex2_rev | ATGGTGTCATTCAGGCGGA | Primer used for RT-qPCR to determine transcription levels of UHRF1 |
| ZNF703_ex1_fw | GTGTCCCTCTTGCCACCG | Primer used for RT-qPCR to determine transcription levels of ZNF703 |
| ZNF703_ex2_rev | AAGGGGCTCTTCTTGGCG | Primer used for RT-qPCR to determine transcription levels of ZNF703 |
| MCM3_ex2_fw | ACCGGCTGATTGTCAATGTG | Primer used for RT-qPCR to determine transcription levels of MCM3 |
| MCM3_ex3_rev | TGCCTTCCAGTCCTACGTAG | Primer used for RT-qPCR to determine transcription levels of MCM3 |
| CGGBP1_ex1_fw | TGCCATTAGTGACCACCTC | Primer used for RT-qPCR to determine transcription levels of CGGBP1 |
| CGGBP1_ex1_rev | TCTCAAGTGGGATGTTGGC | Primer used for RT-qPCR to determine transcription levels of CGGBP1 |
| ABHD5_fw | CCCAGGTTTGACAGTGATGC | Primer used for DRIP-qPCR and RNAPII ChIP-qPCR to determine level of DNA enrichment |
| ABHD5_fw | TGATGGGTACTTCAGCGAGT | Primer used for DRIP-qPCR and RNAPII ChIP-qPCR to determine level of DNA enrichment |
| UBC_fw | TCGTGAAGACTCTGACTGGT | Primer used for DRIP-qPCR and RNAPII ChIP-qPCR to determine level of DNA enrichment |
| UBC_rev | TCCAGCAAAGATCAGCCTCT | Primer used for DRIP-qPCR and RNAPII ChIP-qPCR to determine level of DNA enrichment |
| H2AC7_fw | ATTCAAAATGTCCGGACGCG | Primer used for DRIP-qPCR and RNAPII ChIP-qPCR to determine level of DNA enrichment |
| H2AC7_rev | GCTCGGAGTAGTTGCCCTTG | Primer used for DRIP-qPCR and RNAPII ChIP-qPCR to determine level of DNA enrichment |
| MOV10_fw | CCCTTATGCCAACTACGTGC | Primer used for DRIP-qPCR and RNAPII ChIP-qPCR to determine level of DNA enrichment |
| MOV10_rev | GGGACTCAGTGAAAAGCAGG | Primer used for DRIP-qPCR and RNAPII ChIP-qPCR to determine level of DNA enrichment |
| L3MBTL2_fw | GACTTGGAGCTGTTTGGTGG | Primer used for DRIP-qPCR and RNAPII ChIP-qPCR to determine level of DNA enrichment |
| L3MBTL2_rev | AACCACTGCCATCCAAGGAG | Primer used for DRIP-qPCR and RNAPII ChIP-qPCR to determine level of DNA enrichment |
| Tet-ON_fw | GGCTAGCAAGCTTGATGTG | Primer used for RT-qPCR to determine transcription levels and DRIP enrichment downstream of the (CGG)_10_ repeat on plasmid pHU |
| Tet-ON_rev | GCAATAGCATCACAAATTTCACA | Primer used for RT-qPCR to determine transcription levels and DRIP enrichment downstream of the (CGG)_10_ repeat on plasmid pHU |
| OriP_fw | TTTTCGCTGCTTGTCCTTTT | Primer used for qPCR to determine plasmid copy number |
| OriP_rev | TTTTCGCTGCTTGTCCTTTT | Primer used for qPCR to determine plasmid copy number |
| β-actin_in1_fw | CGGGGTCTTTGTCTGAGC | Primer used for qPCR to determine genomic β-actin DNA amount as reference |
| β-actin_in1_rev | CAGTTAGCGCCCAAAGGAC | Primer used for qPCR to determine genomic β-actin DNA amount as reference |
| TSS_C7orf50_fwd | CATTAGCCGGCGGAGAGA | Primer used for RNAPII ChIP-qPCR to determine level of DNA enrichment |
| TSS_C7orf50_rev | CCACGTCGCCCACCTAAG | Primer used for RNAPII ChIP-qPCR to determine level of DNA enrichment |
| TSS_EGR1_fwd | TATGGCCATGTACGTCACGA | Primer used for RNAPII ChIP-qPCR to determine level of DNA enrichment |
| TSS_EGR1_rev | GGCTCCCCAAGTTCTGCG | Primer used for RNAPII ChIP-qPCR to determine level of DNA enrichment |
| TSS_EF1F3_fwd | AAAGCAGTTGAGTTTGGGGC | Primer used for RNAPII ChIP-qPCR to determine level of DNA enrichment |
| TSS_EF1F3_rev | CAATCCCCACCTTGCCTTTC | Primer used for RNAPII ChIP-qPCR to determine level of DNA enrichment |
| TSS_SEC22B_fwd | TTGCTAACAATGATCGCCCG | Primer used for RNAPII ChIP-qPCR to determine level of DNA enrichment |
| TSS_SEC22B_rev | CTTCAGAGAGGTCGGGAGTG | Primer used for RNAPII ChIP-qPCR to determine level of DNA enrichment |
| TSS_RPS29_fwd | CACTTCACGCACCTCATAGC | Primer used for RNAPII ChIP-qPCR to determine level of DNA enrichment |
| TSS_RPS29_rev | TGTGAGGTGGAGGTGATCAG | Primer used for RNAPII ChIP-qPCR to determine level of DNA enrichment |
| TSS_IRFB2P_fwd | GGAAGTGTAAAGGGGAGGGG | Primer used for RNAPII ChIP-qPCR to determine level of DNA enrichment |
| TSS_IRF2B_rev | AGTGGTCCTTCTTGAAGCG | Primer used for RNAPII ChIP-qPCR to determine level of DNA enrichment |
| GB_C7orf50_fwd | ACGTAGCTTTGTTCCCTCCA | Primer used for RNAPII ChIP-qPCR to determine level of DNA enrichment |
| GB_C7orf50_rev | ACCATGCAAAGACATCCACG | Primer used for RNAPII ChIP-qPCR to determine level of DNA enrichment |
| GB_EGR1_fwd | CTTCCCTTCCTCAGCTGTCA | Primer used for RNAPII ChIP-qPCR to determine level of DNA enrichment |
| GB_EGR1_rev | GAACCCTCCTCTCCTATGGC | Primer used for RNAPII ChIP-qPCR to determine level of DNA enrichment |
| GB_SEC22B_fwd | TCAAGCGATCTTCCCACCTT | Primer used for RNAPII ChIP-qPCR to determine level of DNA enrichment |
| GB_SEC22B_rev | TTGAGCCCTGGAGTTTGTGA | Primer used for RNAPII ChIP-qPCR to determine level of DNA enrichment |
| GB_RPS29_fwd | ATATGTGCCGCCAGTGTTTC | Primer used for RNAPII ChIP-qPCR to determine level of DNA enrichment |
| GB_RPS29_rev | GCTCTTGGTCGAATGCTCAG | Primer used for RNAPII ChIP-qPCR to determine level of DNA enrichment |
| GB_IRFB2P_fwd | GAGTTGAAGGAGAAGCAGCG | Primer used for RNAPII ChIP-qPCR to determine level of DNA enrichment |
| GB_IRF2B_rev | AGTGGTCCTTCTTGAAGCGA | Primer used for RNAPII ChIP-qPCR to determine level of DNA enrichment |
