# Supplementary figures and images for: The CGG triplet repeat binding protein 1 counteracts R-loop induced transcription-replication stress
Source: EMBO Rep. 2025 Aug 26;26(19):4691–722. doi: 10.1038/s44319-025-00550-1 (PMC12508481; doi:10.1038/s44319-025-00550-1)

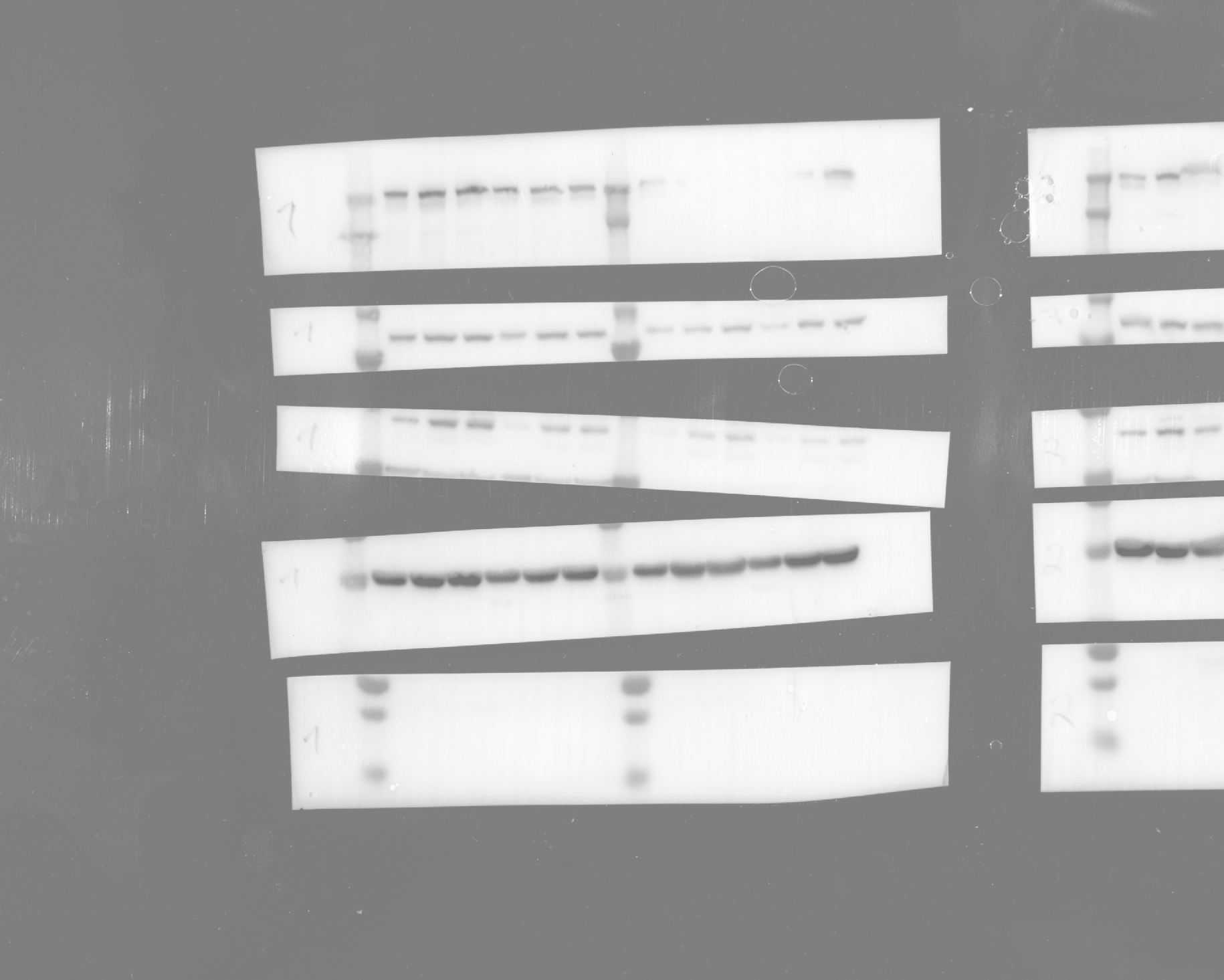

Supplement: Supplementary file 3 — Source data Fig. 2 [file 44319_2025_550_MOESM3_ESM.zip › Figure 2/2A/2022-04-02 13h18m08s Chemiluminescence 18.814s Colorimetric 0.342s(Composite).tif]

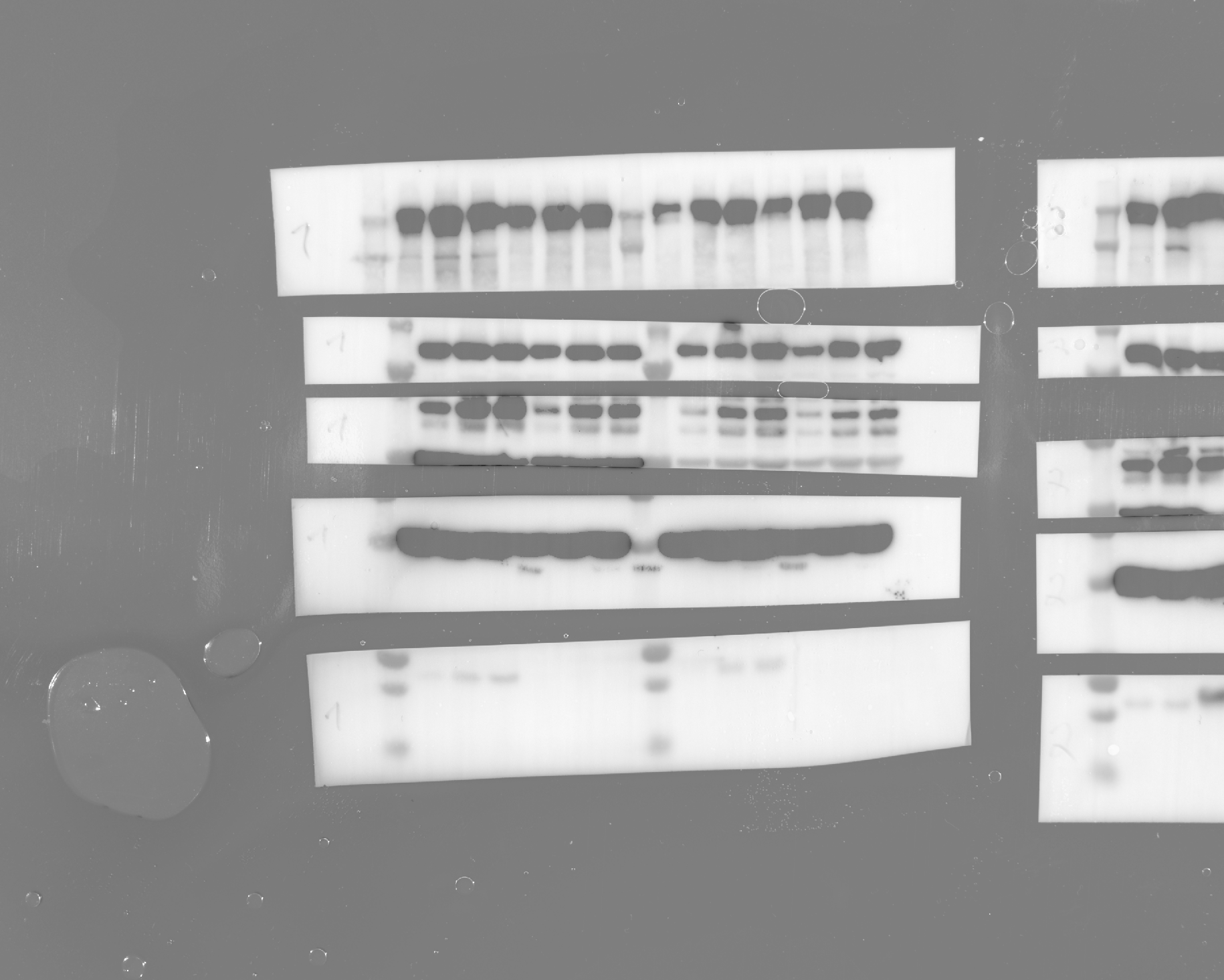

Supplement: Supplementary file 3 — Source data Fig. 2 [file 44319_2025_550_MOESM3_ESM.zip › Figure 2/2A/2022-04-02 13h29m17s Chemiluminescence 200.000s Colorimetric 0.335s(Composite).tif]

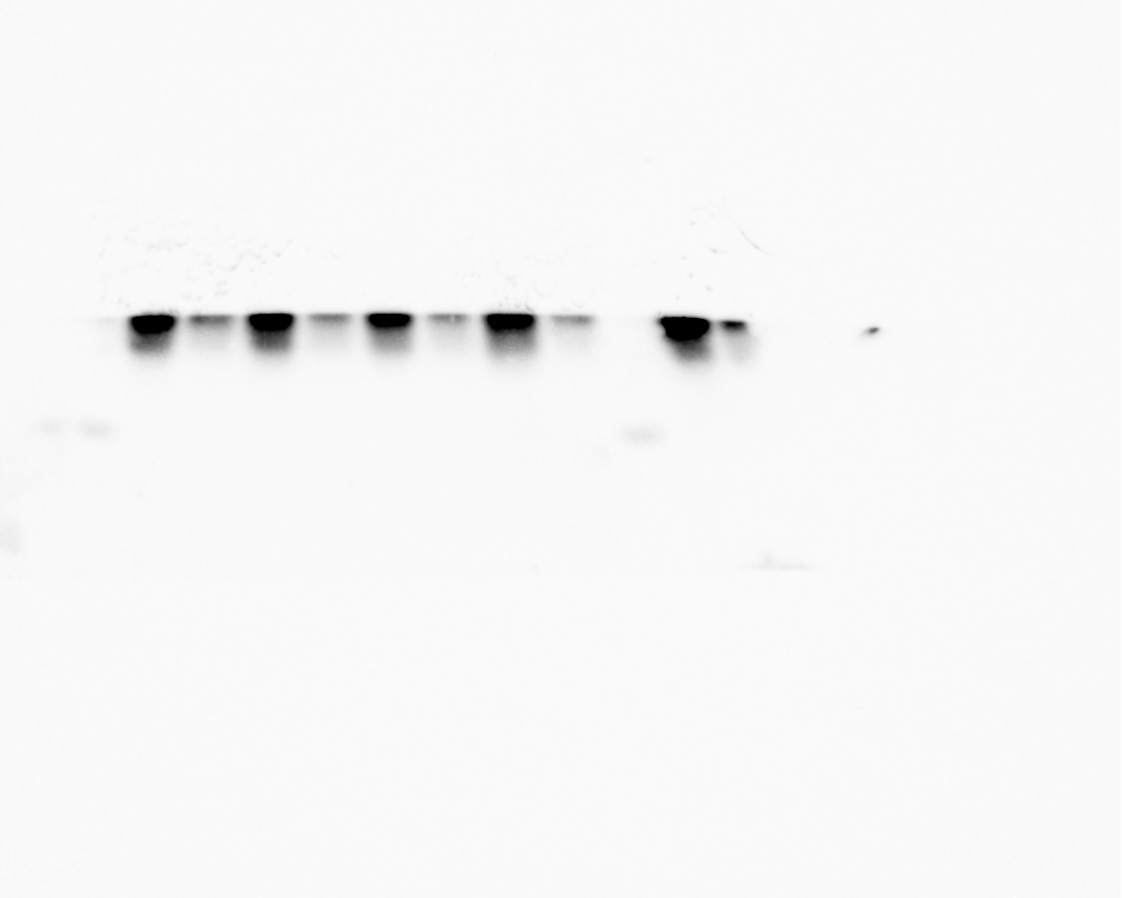

Supplement: Supplementary file 10 — Source data Fig. 4 [file 44319_2025_550_MOESM10_ESM.zip › Figure 4/4J/4J CGGBP1.tif]

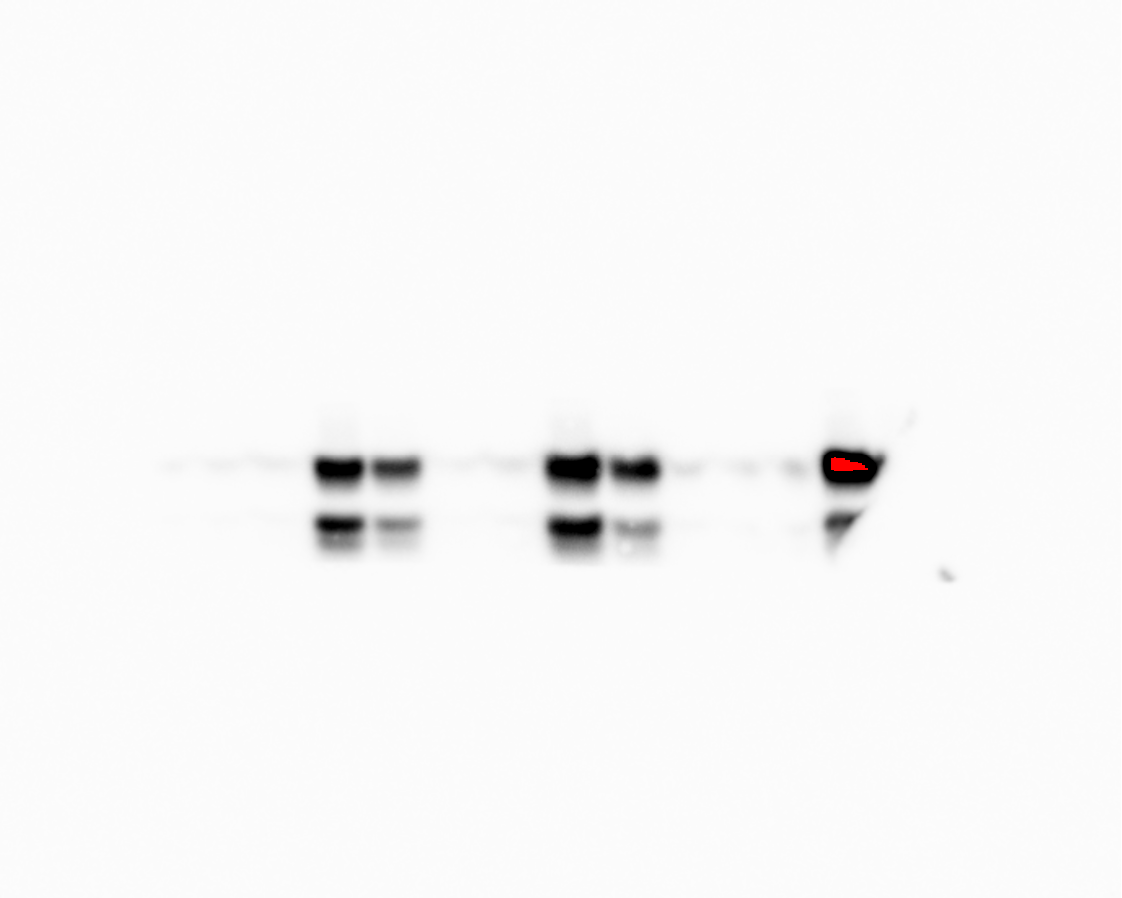

Supplement: Supplementary file 10 — Source data Fig. 4 [file 44319_2025_550_MOESM10_ESM.zip › Figure 4/4J/4J FLAG.tif]

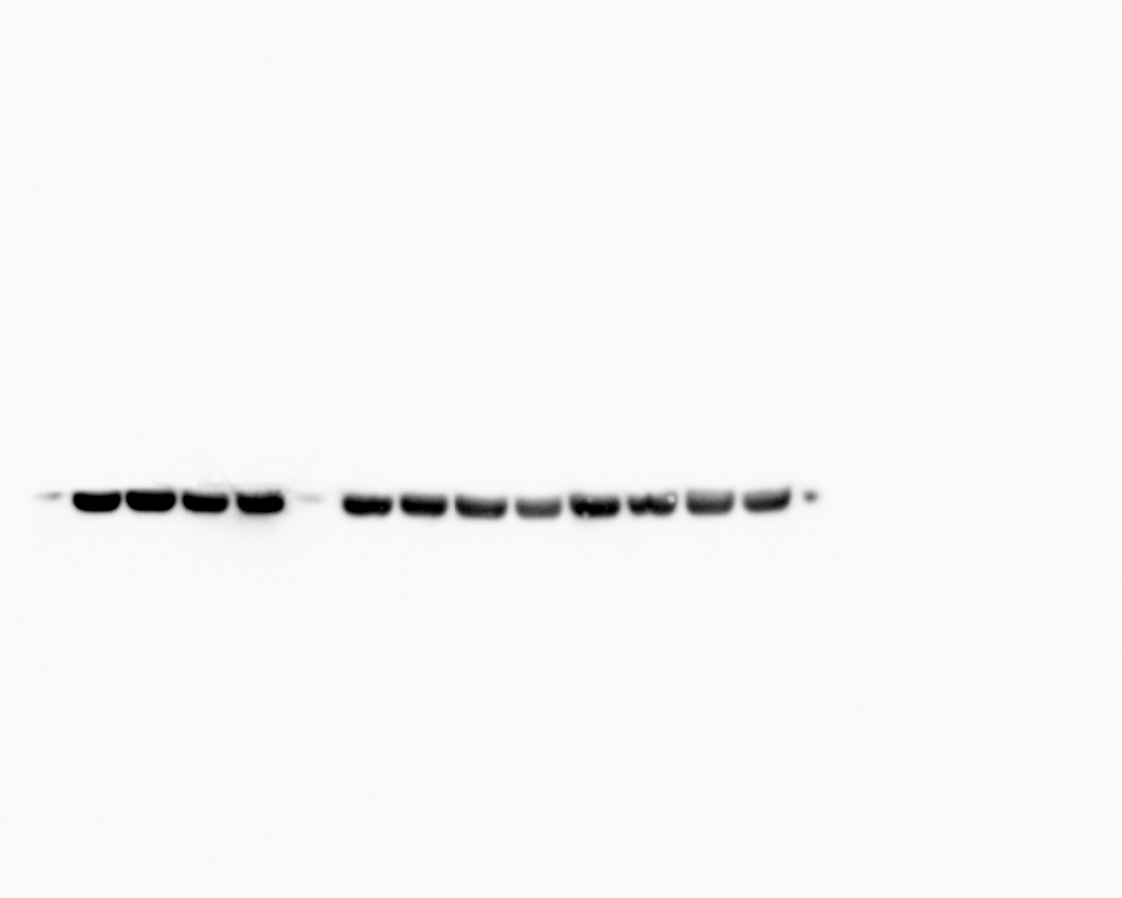

Supplement: Supplementary file 10 — Source data Fig. 4 [file 44319_2025_550_MOESM10_ESM.zip › Figure 4/4J/4J GAPDH.tif]

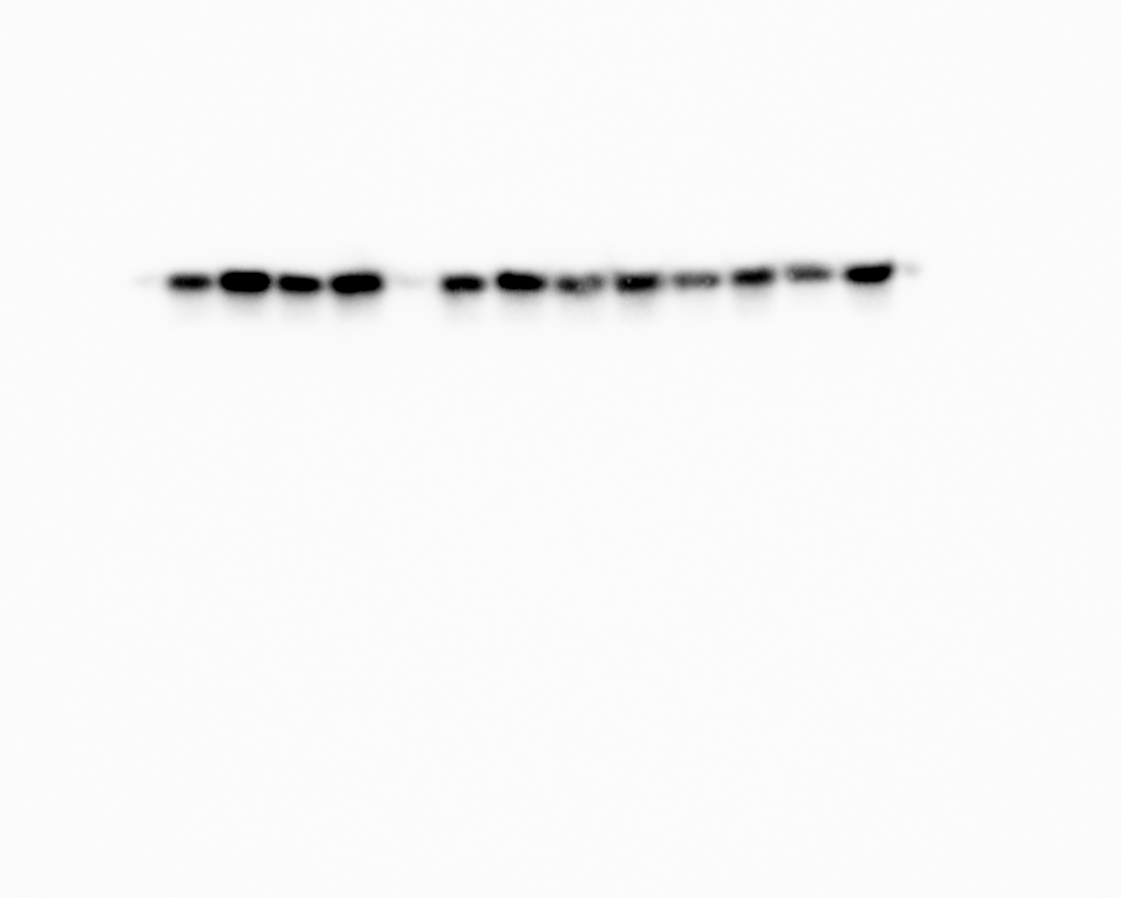

Supplement: Supplementary file 10 — Source data Fig. 4 [file 44319_2025_550_MOESM10_ESM.zip › Figure 4/4J/4J yH2AX.tif]

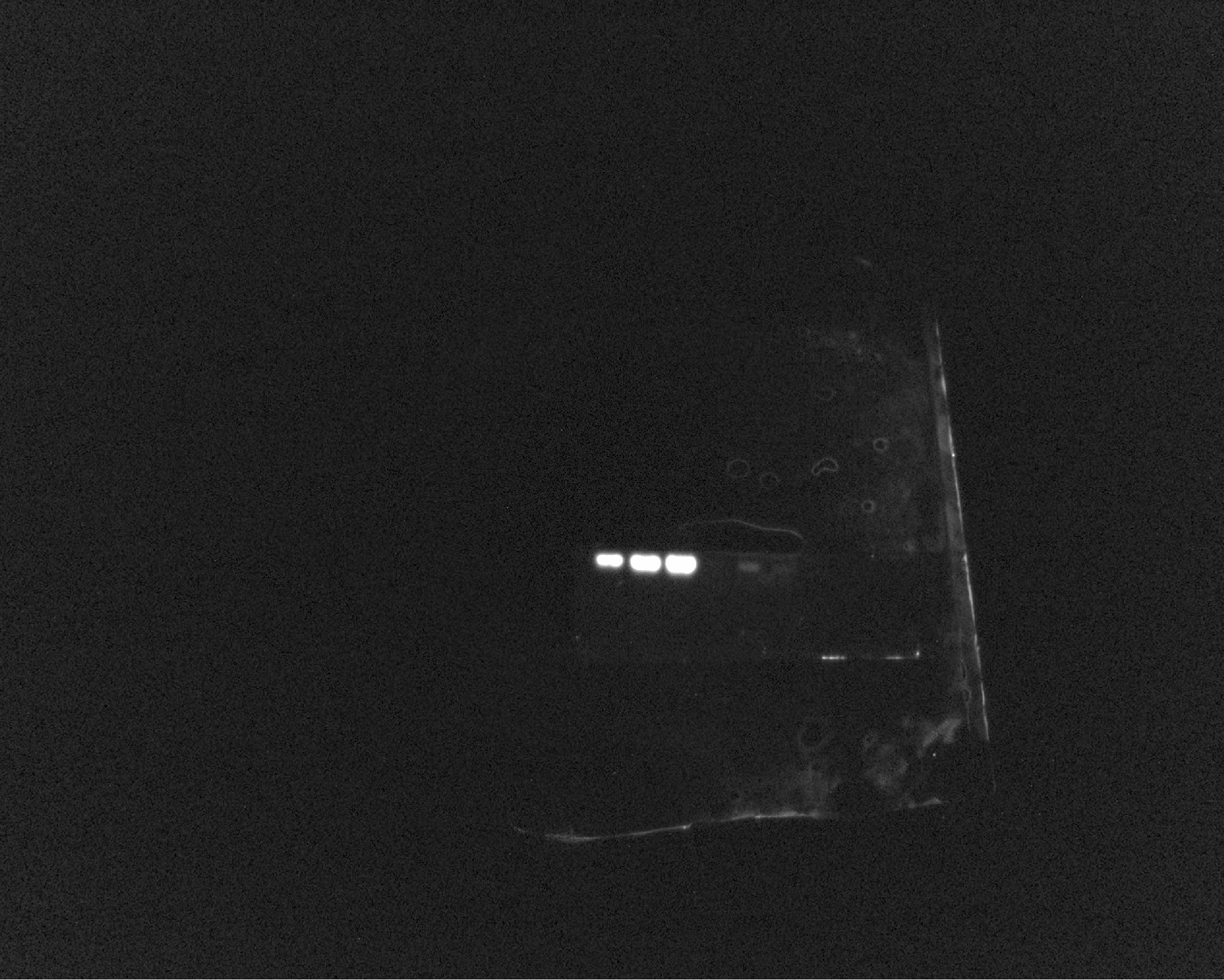

Supplement: Supplementary file 11 — Source data Fig. 5 [file 44319_2025_550_MOESM11_ESM.zip › Figure 5/5B/CGGBP1.tif]

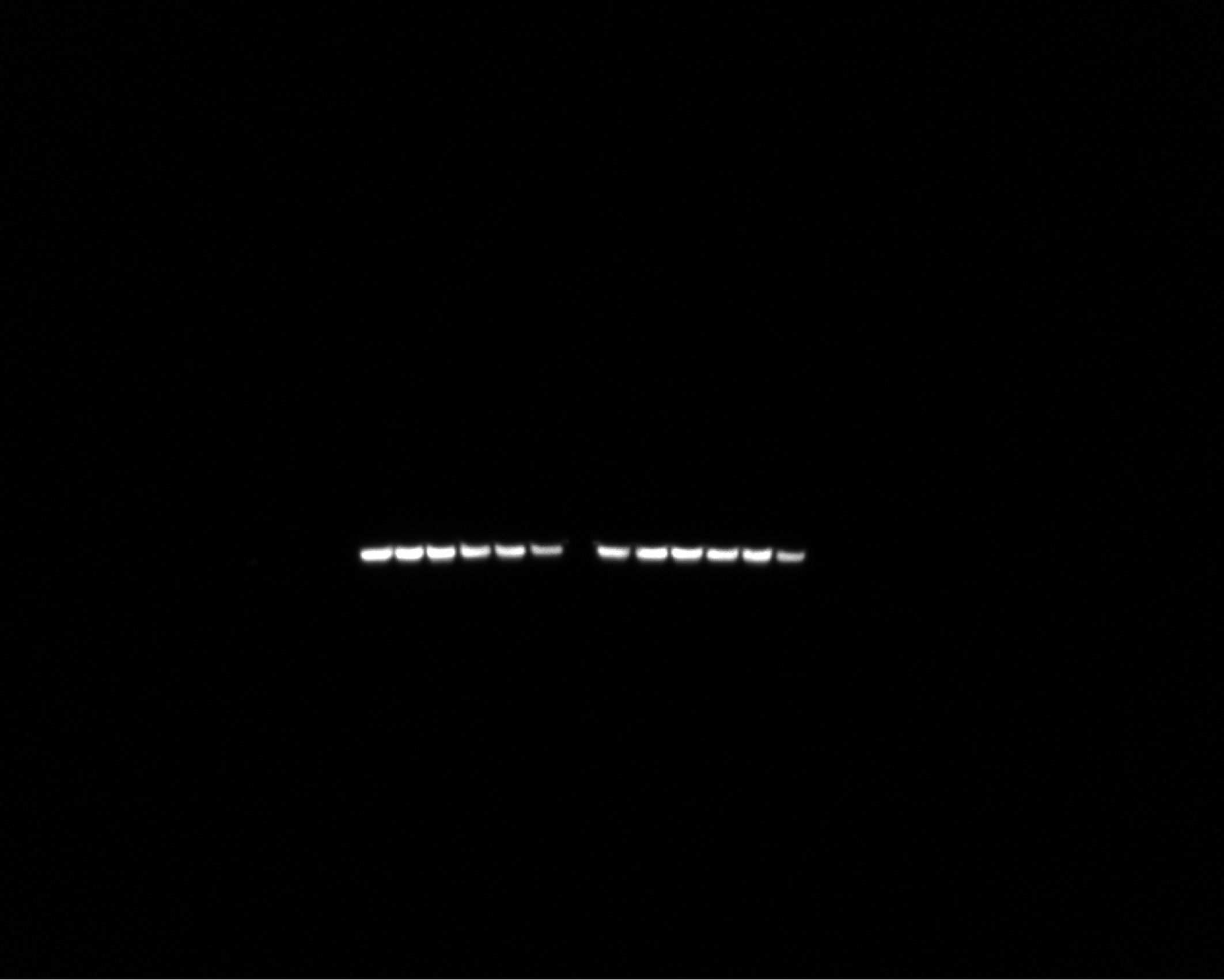

Supplement: Supplementary file 11 — Source data Fig. 5 [file 44319_2025_550_MOESM11_ESM.zip › Figure 5/5B/GAPDH.tif]

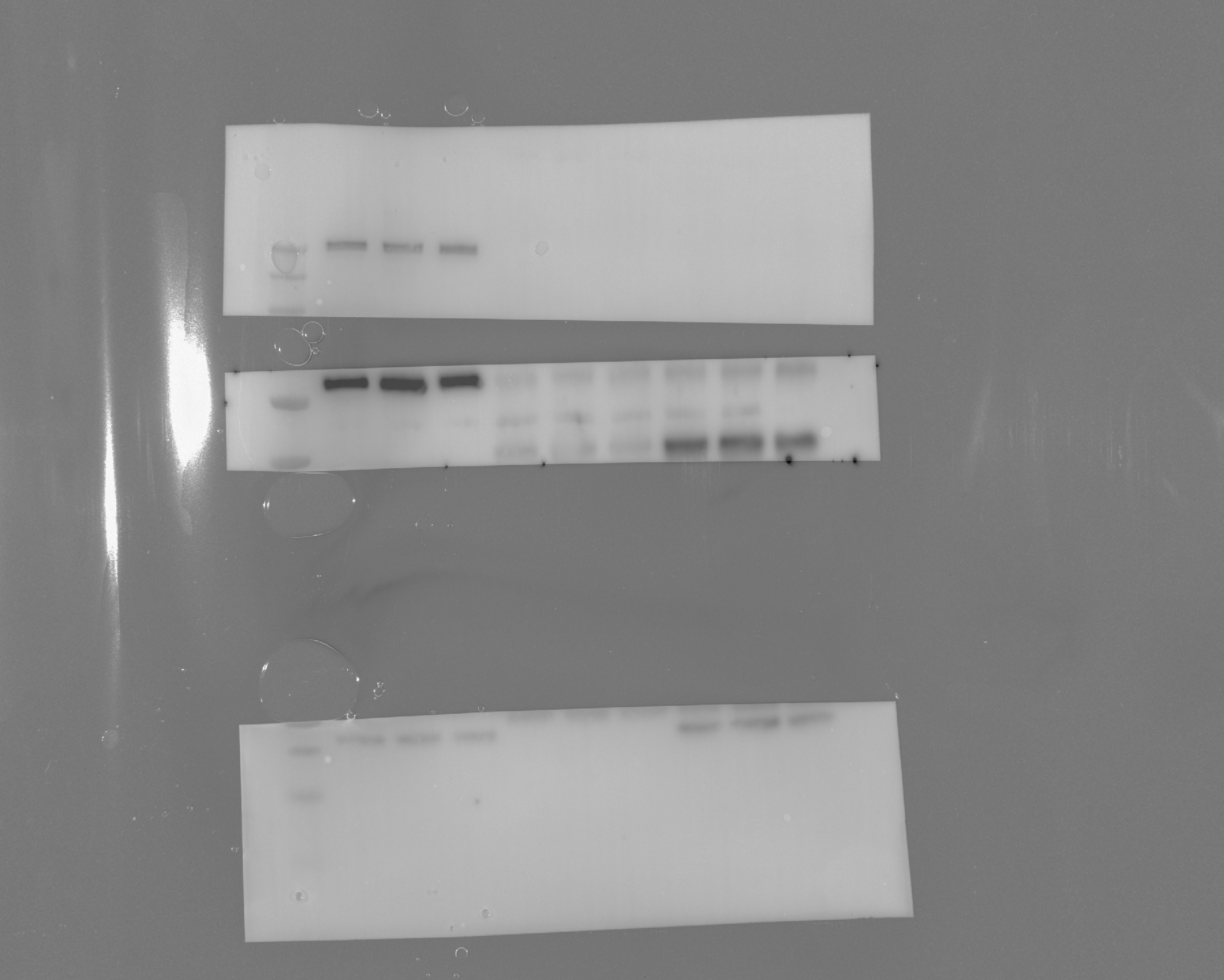

Supplement: Supplementary file 12 — Source data Fig. 6 [file 44319_2025_550_MOESM12_ESM.zip › Figure 6/6A/CGGBP1.tif]

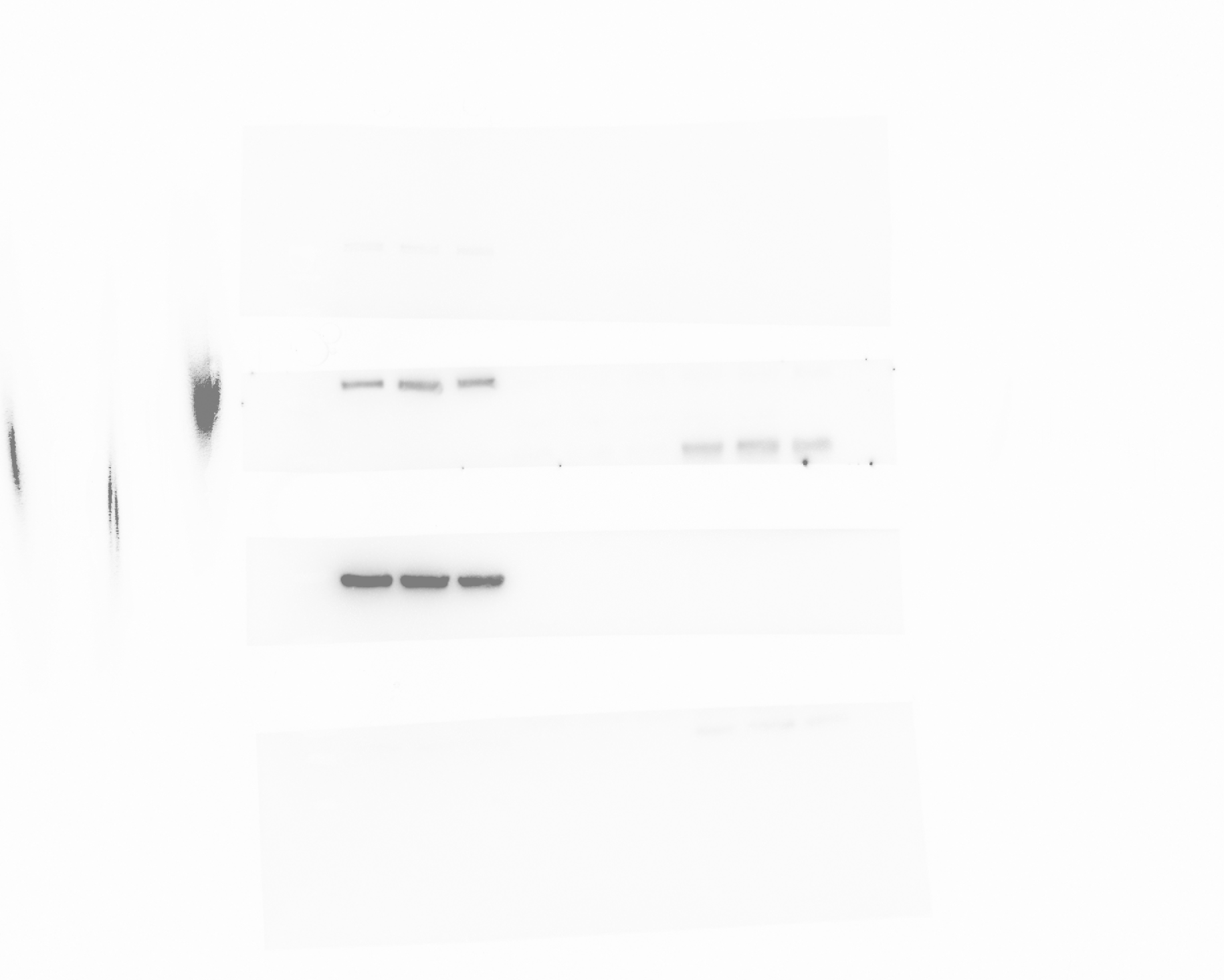

Supplement: Supplementary file 12 — Source data Fig. 6 [file 44319_2025_550_MOESM12_ESM.zip › Figure 6/6A/GAPDH.tif]

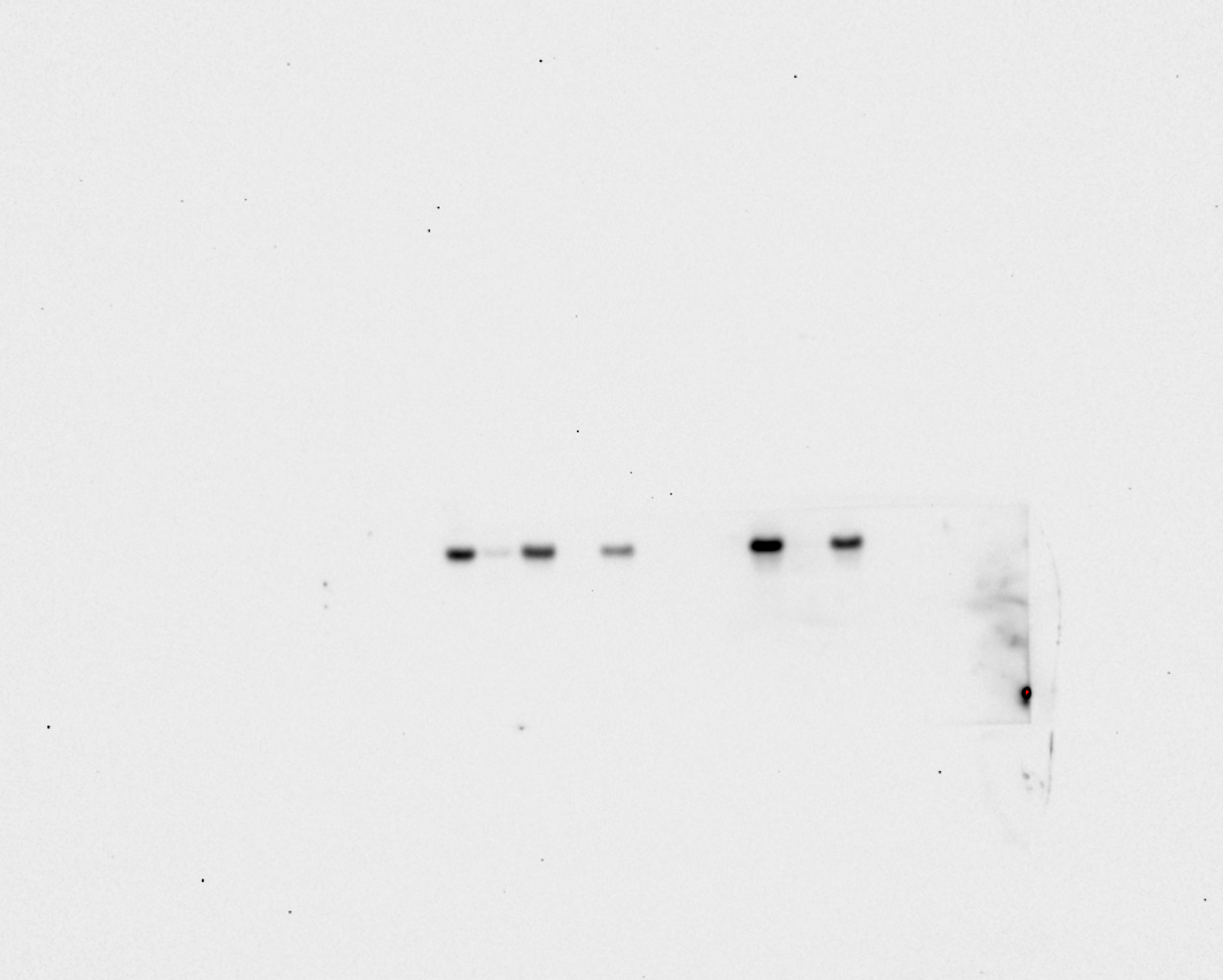

Supplement: Supplementary file 13 — Source data Fig. 7 [file 44319_2025_550_MOESM13_ESM.zip › Figure 7/7A/CGGBP1 for DDX41 knockdown(Chemiluminescence).tif]

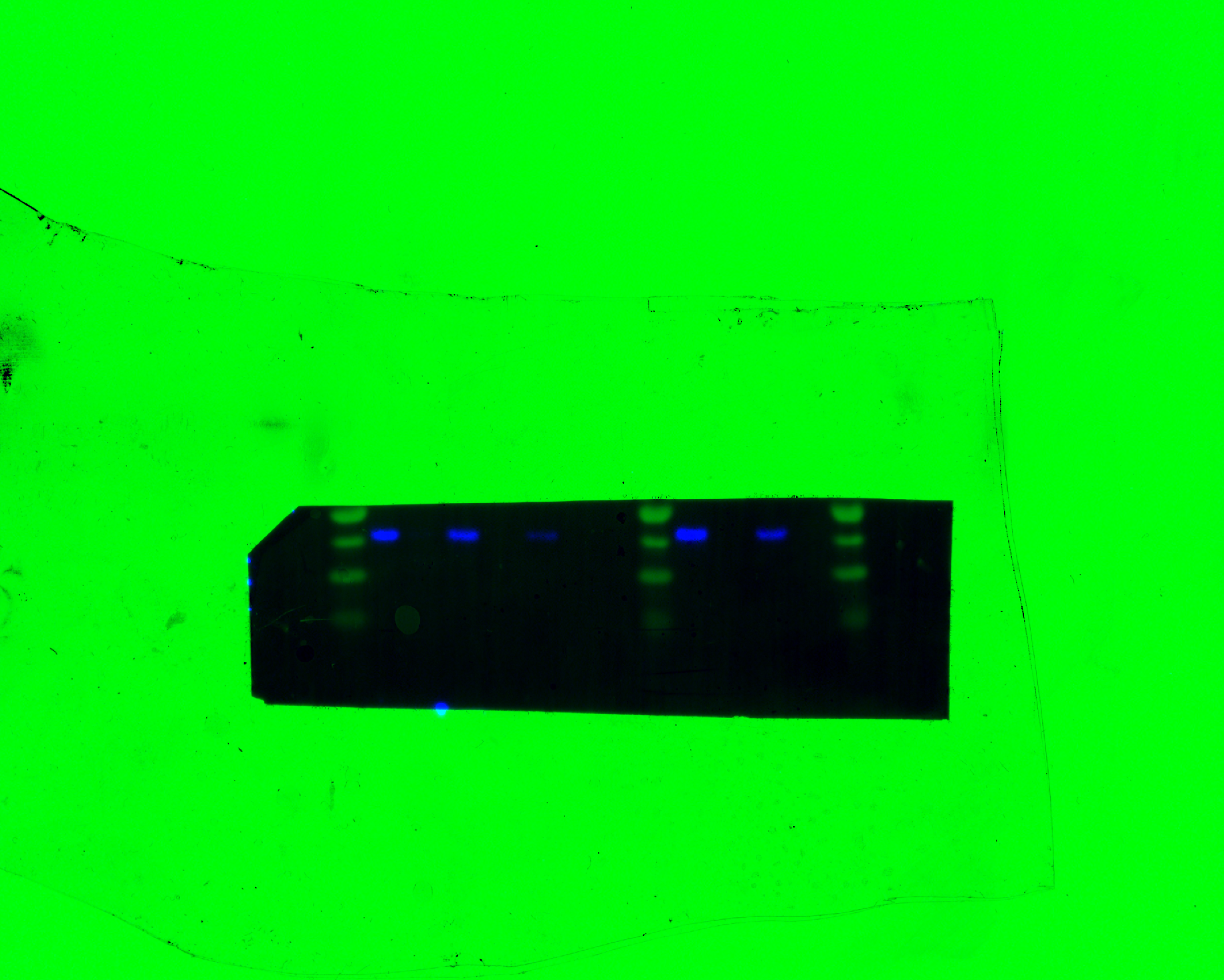

Supplement: Supplementary file 13 — Source data Fig. 7 [file 44319_2025_550_MOESM13_ESM.zip › Figure 7/7A/CGGBP1 for DDX41 knockdwon(Composite).tif]

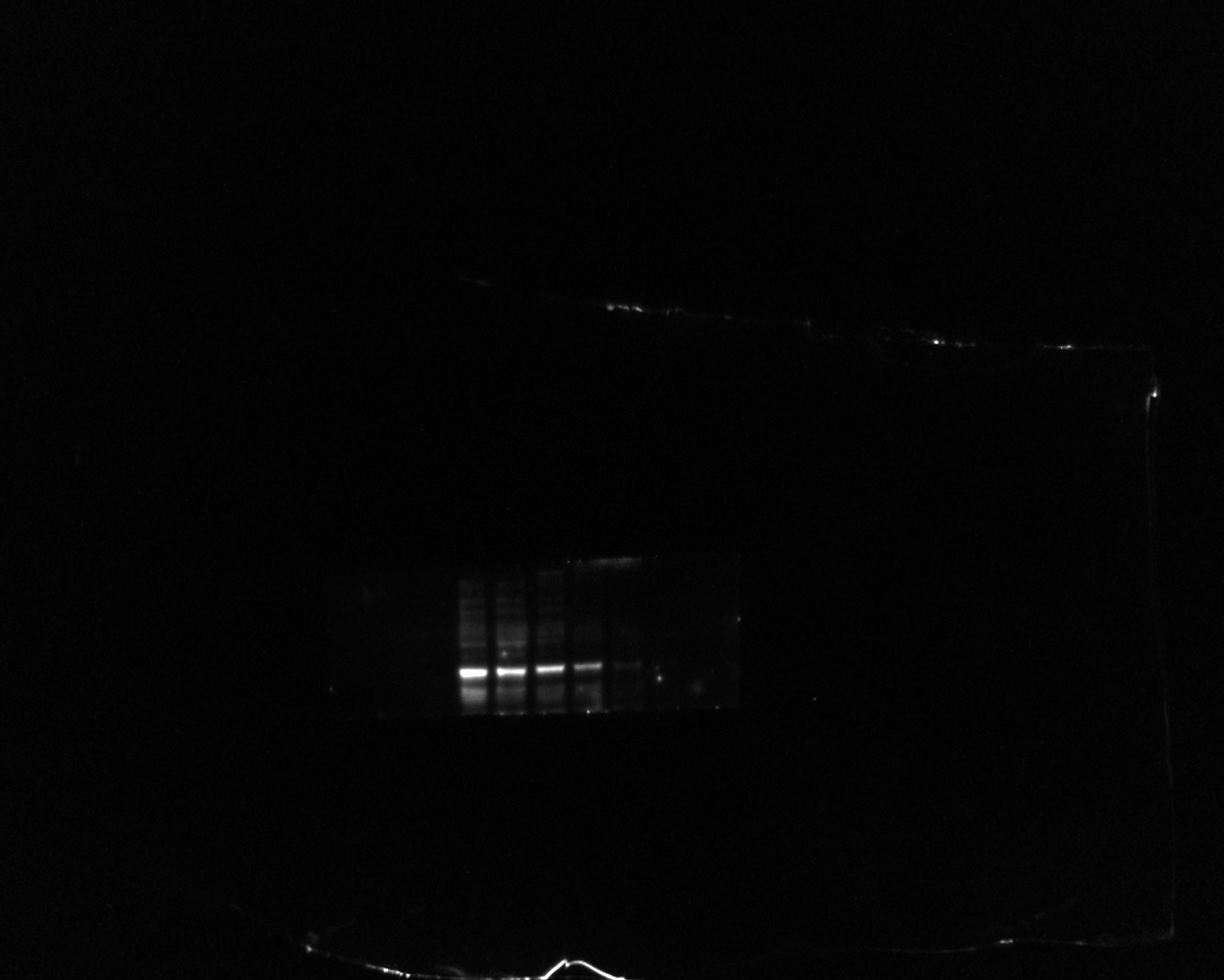

Supplement: Supplementary file 13 — Source data Fig. 7 [file 44319_2025_550_MOESM13_ESM.zip › Figure 7/7A/DDX41 knockdown(Chemiluminescence).tif]

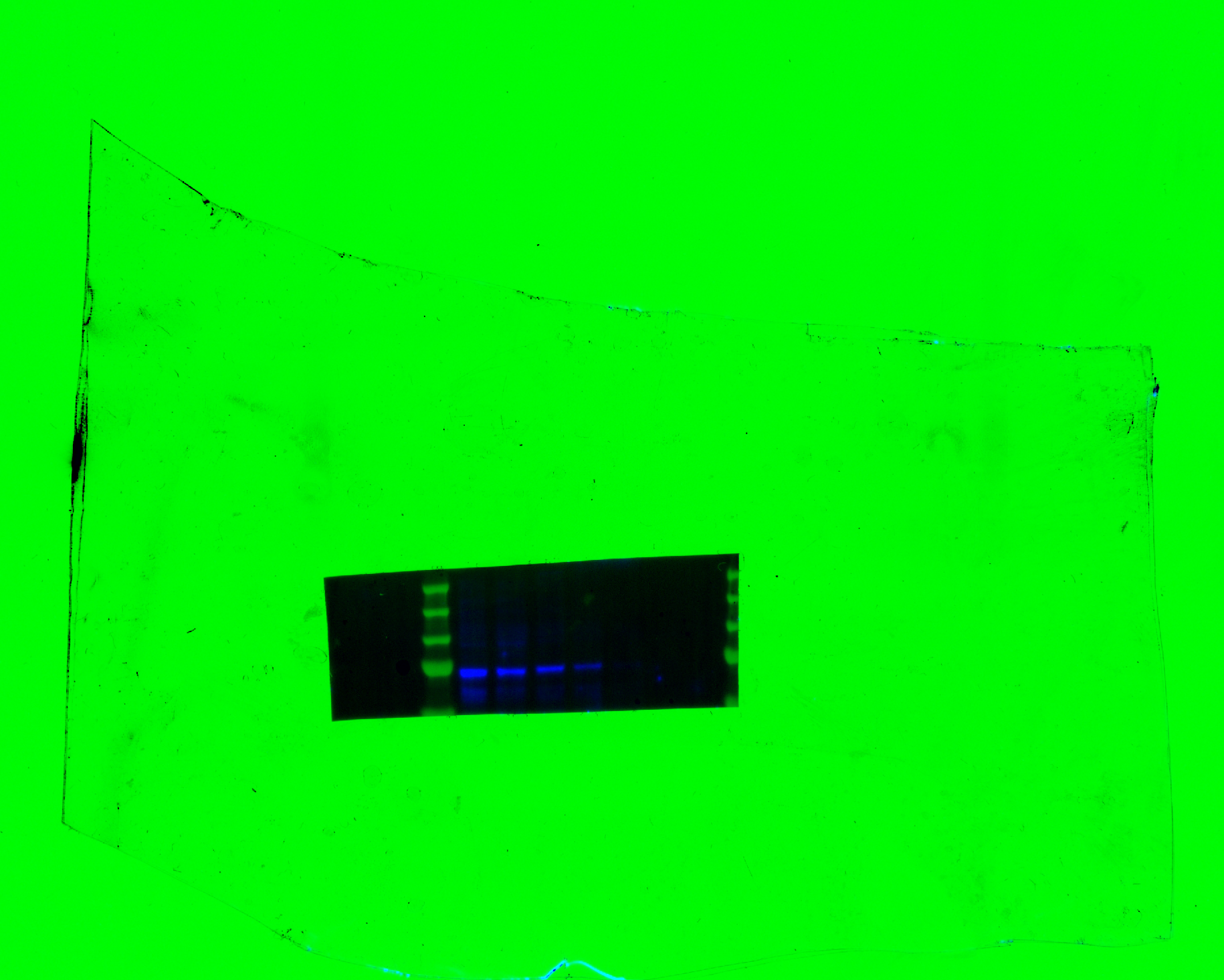

Supplement: Supplementary file 13 — Source data Fig. 7 [file 44319_2025_550_MOESM13_ESM.zip › Figure 7/7A/DDX41 knockdown(Composite).tif]

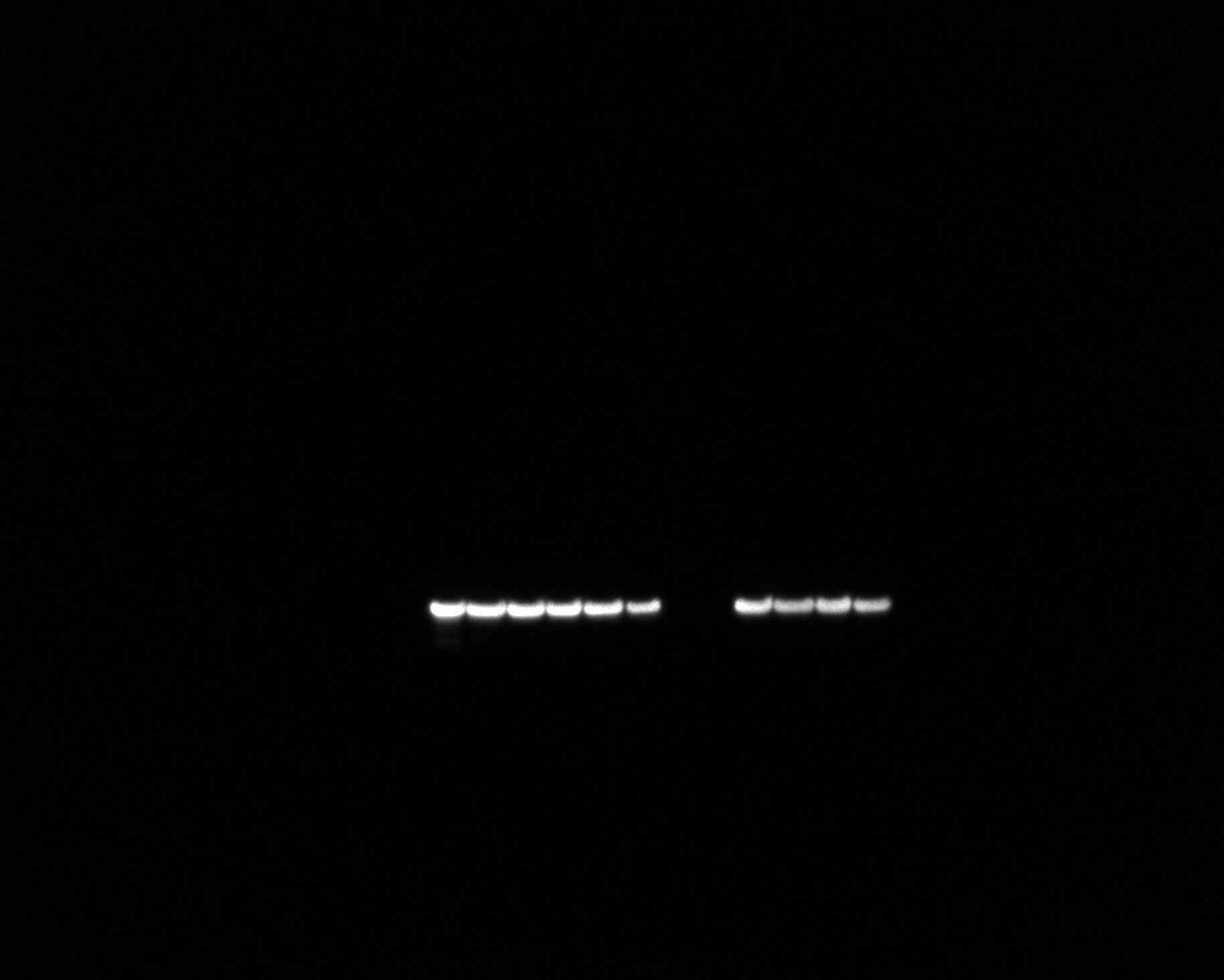

Supplement: Supplementary file 13 — Source data Fig. 7 [file 44319_2025_550_MOESM13_ESM.zip › Figure 7/7A/gapdh83(Chemiluminescence).tif]

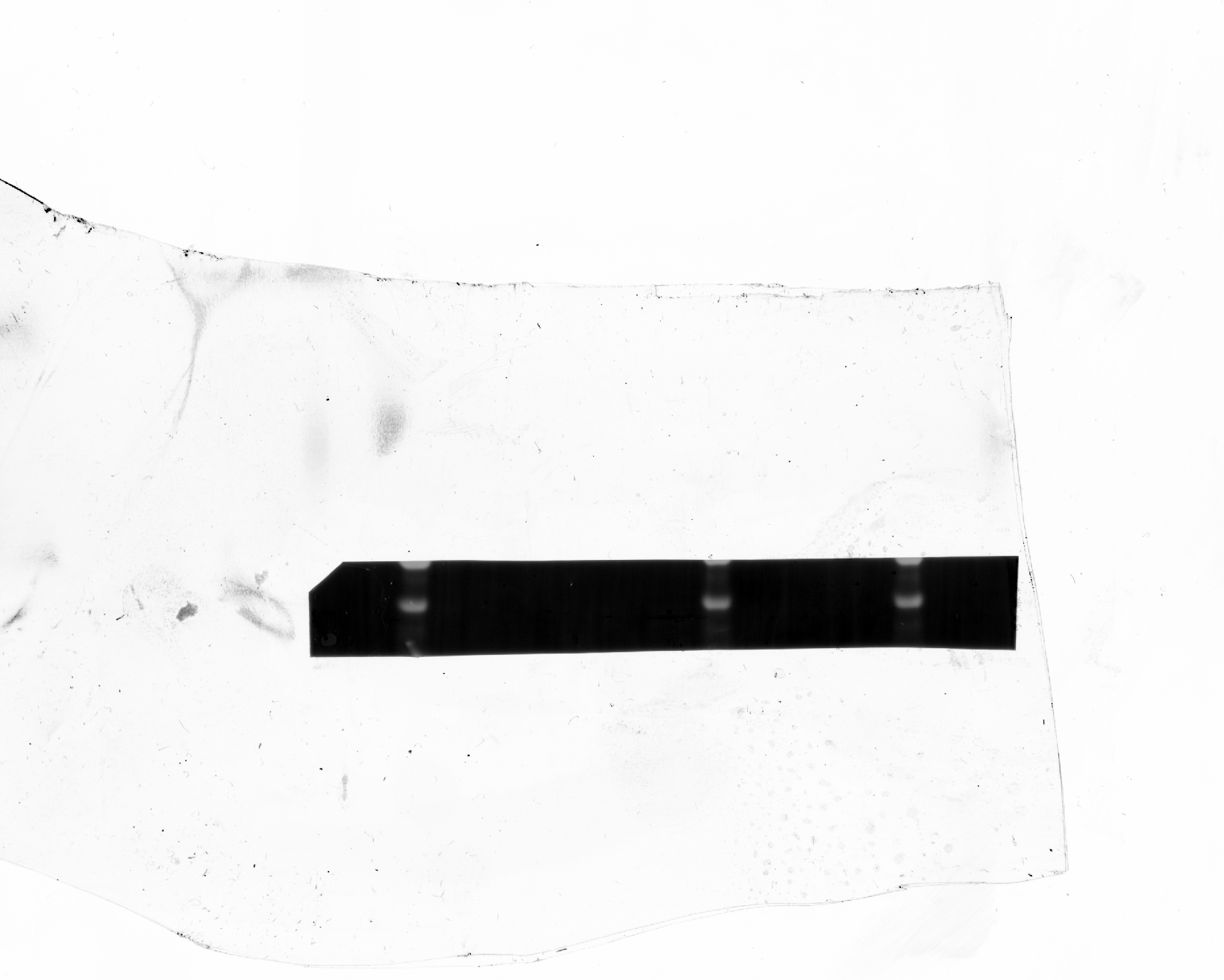

Supplement: Supplementary file 13 — Source data Fig. 7 [file 44319_2025_550_MOESM13_ESM.zip › Figure 7/7A/gapdh83(Colorimetric).tif]

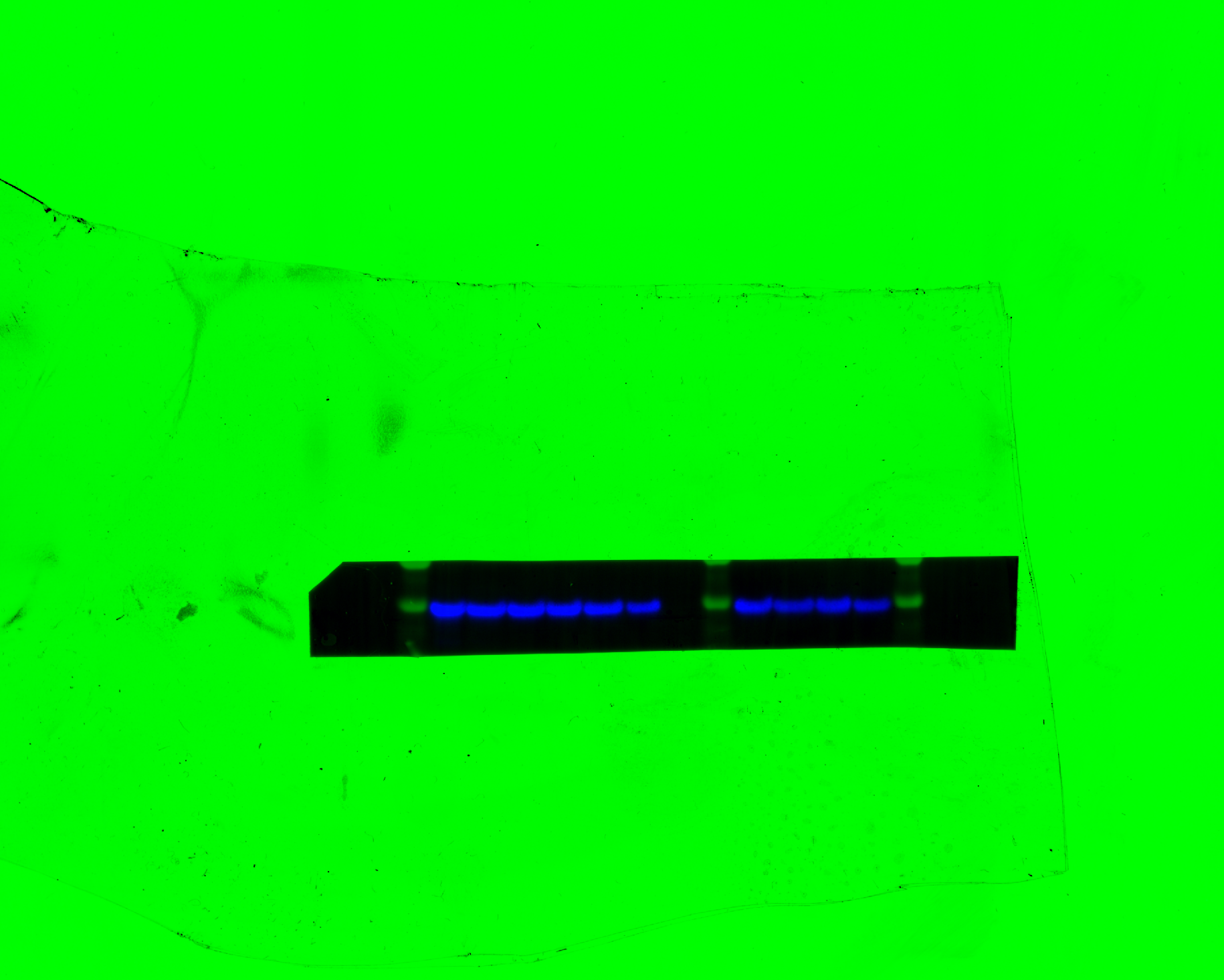

Supplement: Supplementary file 13 — Source data Fig. 7 [file 44319_2025_550_MOESM13_ESM.zip › Figure 7/7A/gapdh83(Composite).tif]

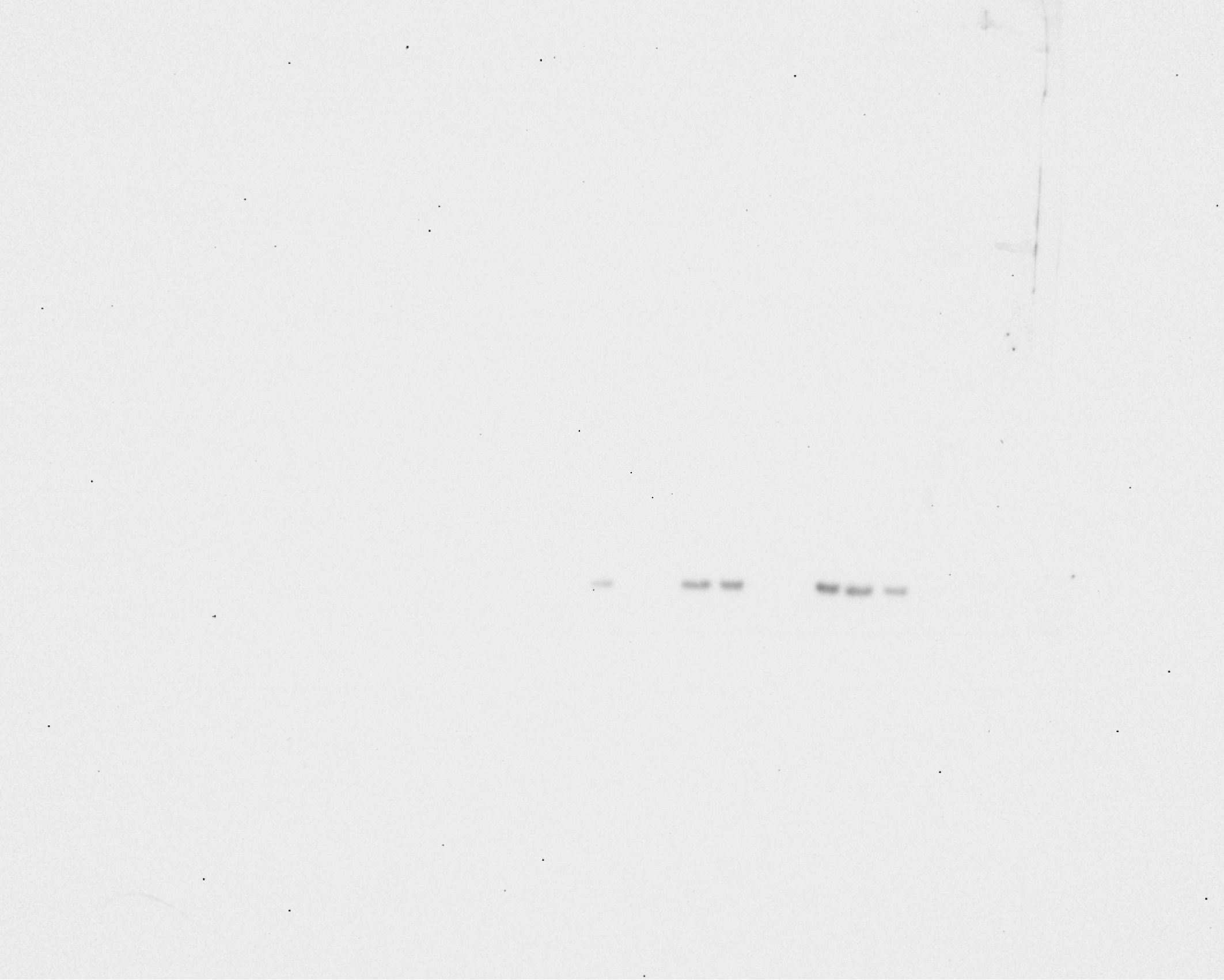

Supplement: Supplementary file 13 — Source data Fig. 7 [file 44319_2025_550_MOESM13_ESM.zip › Figure 7/7B/dhx15cggbp1_6(Chemiluminescence).tif]

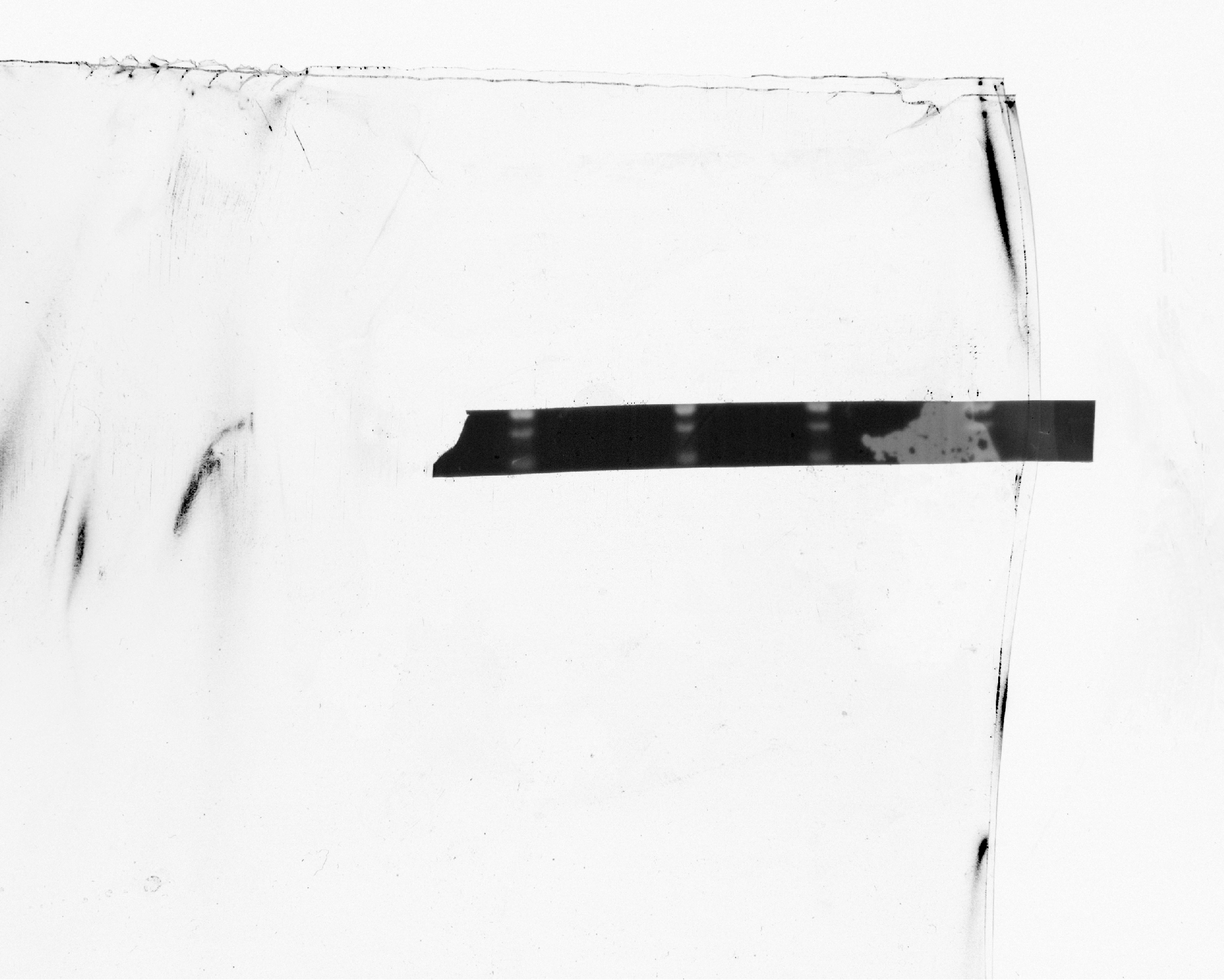

Supplement: Supplementary file 13 — Source data Fig. 7 [file 44319_2025_550_MOESM13_ESM.zip › Figure 7/7B/dhx15ggbp1(Colorimetric).tif]

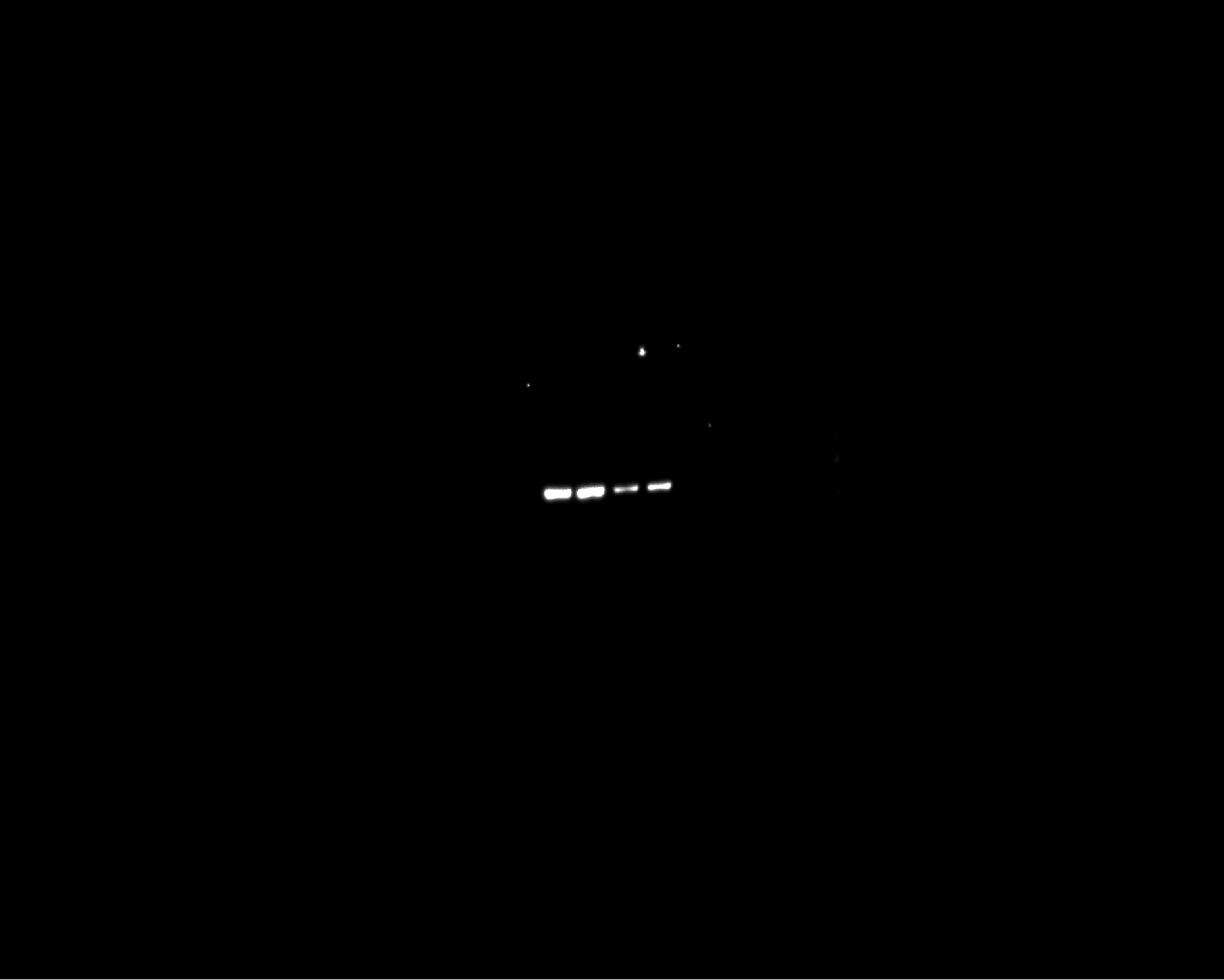

Supplement: Supplementary file 13 — Source data Fig. 7 [file 44319_2025_550_MOESM13_ESM.zip › Figure 7/7B/DHX15_knockdown(Chemiluminescence).tif]

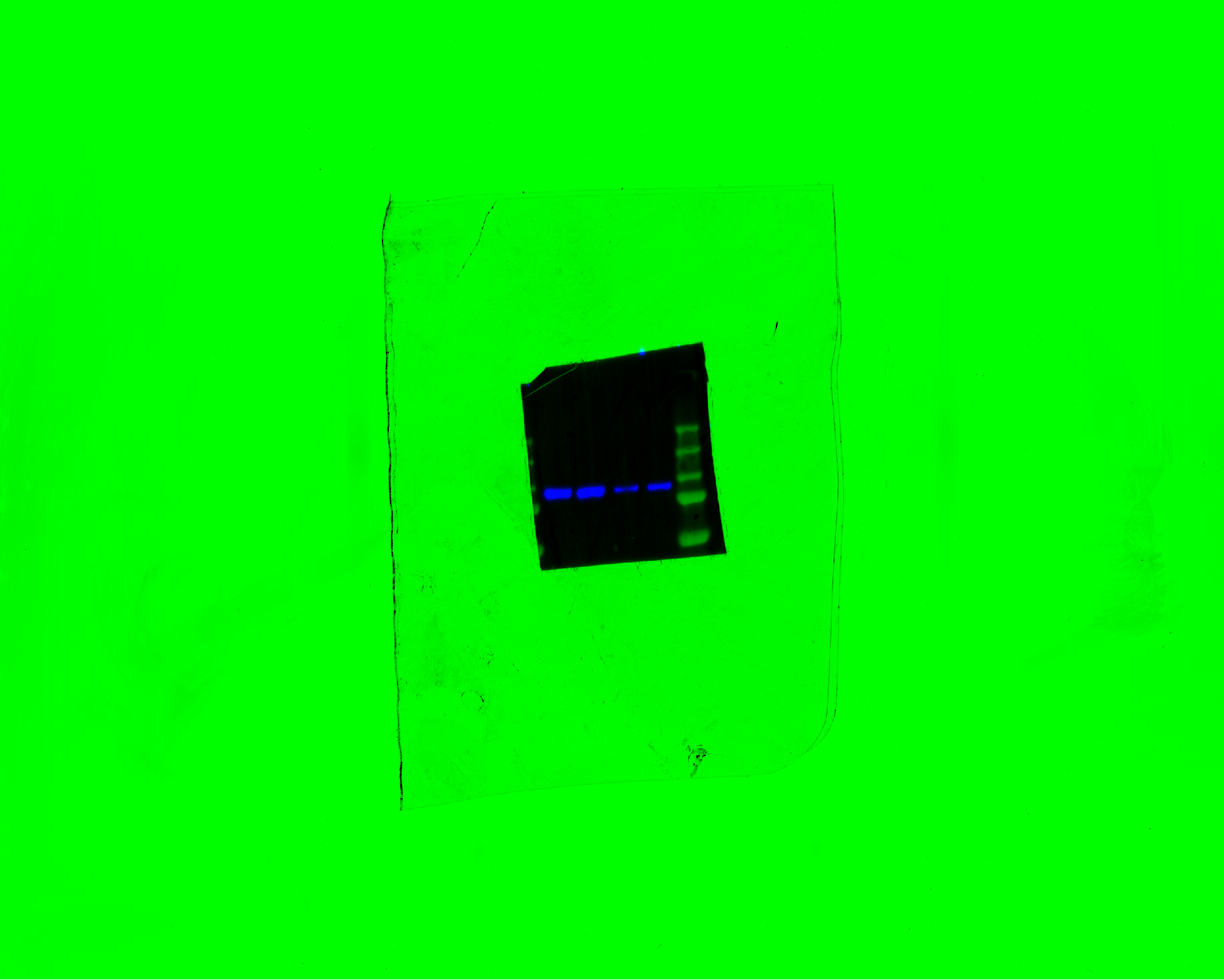

Supplement: Supplementary file 13 — Source data Fig. 7 [file 44319_2025_550_MOESM13_ESM.zip › Figure 7/7B/DHX15_knockdown(Composite).tif]

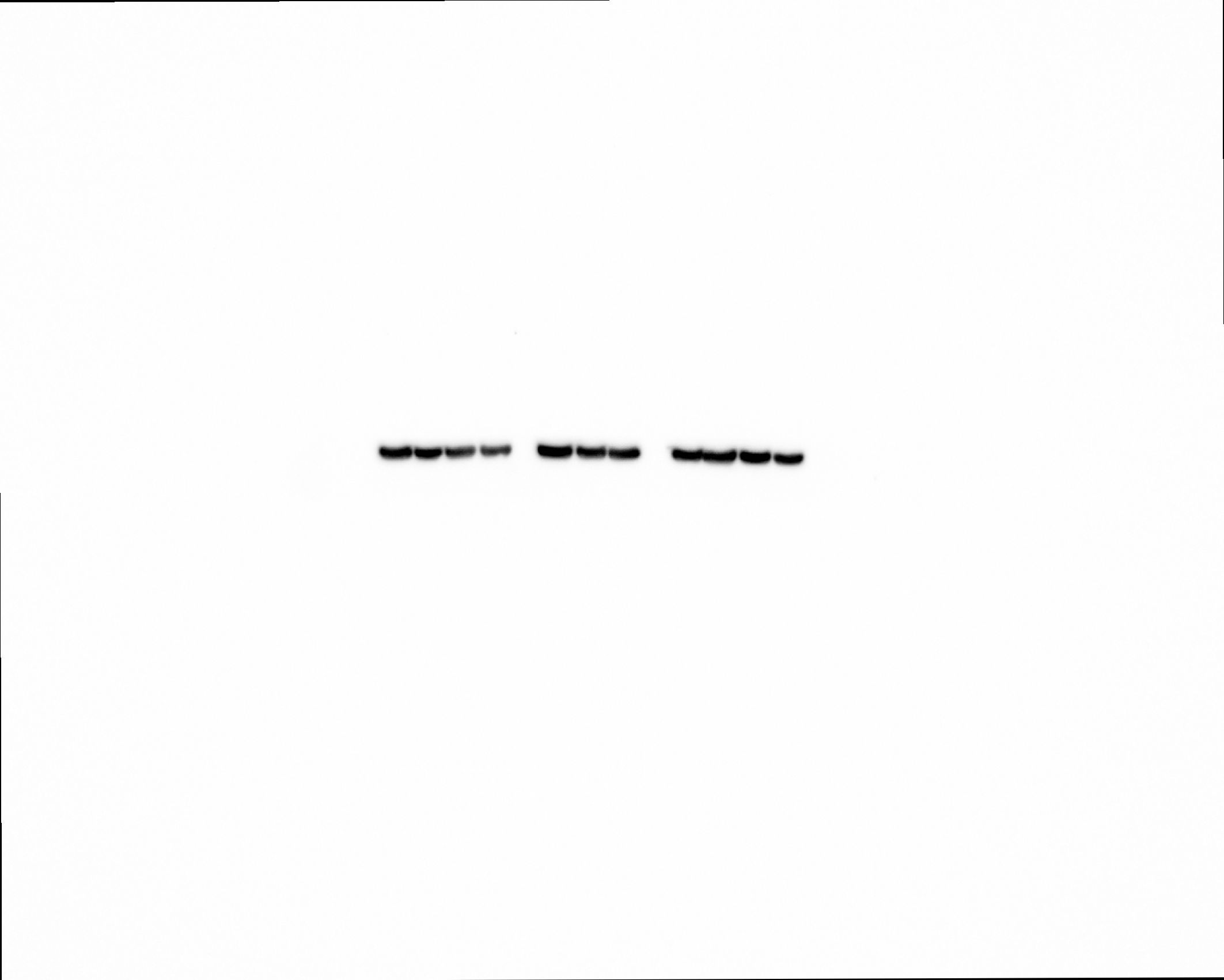

Supplement: Supplementary file 13 — Source data Fig. 7 [file 44319_2025_550_MOESM13_ESM.zip › Figure 7/7B/GAPDHforDH15knockdown.tif]
