# Supplementary material for: The CGG triplet repeat binding protein 1 counteracts R-loop induced transcription-replication stress
Source: EMBO Rep. 2025 Aug 26;26(19):4691–722. doi: 10.1038/s44319-025-00550-1 (PMC12508481; doi:10.1038/s44319-025-00550-1)
Supplement: Supplementary file 14 — Expanded View Figures [file 44319_2025_550_MOESM14_ESM.pdf]

Expanded View Figures

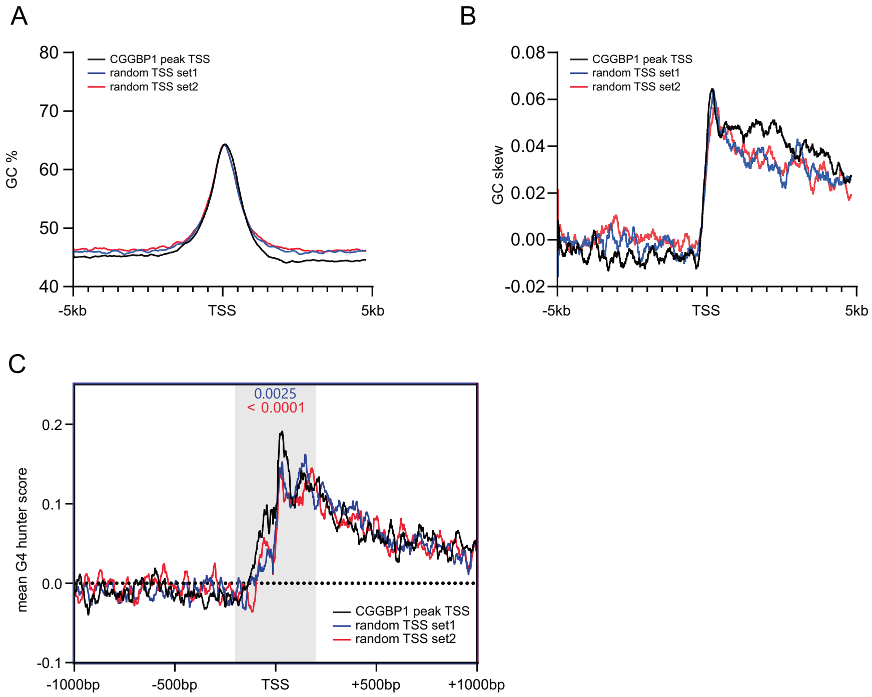

Figure EV1. Global profiling of CGGBP1 binding sites in the human genome.

(**A**) Summary plot of GC% of the DNA sequence at either TSSs with a CGGBP1 binding peak in proximity or two random sets of TSSs. (**B**) Summary plot of GC skew of the DNA sequence at either TSSs with a CGGBP1 binding peak in proximity or two random sets of TSSs. GC skew was calculated with the formula (G–C)/(G + C). (**C**) Summary plot of predicted G4 quadruplex formation scores at either TSSs with a CGGBP1 binding peak in proximity or two random sets of TSSs. Statistical significance was calculated using two-tailed Student's *t* test on the medians of 10 bp windows at the TSS +/− 100 bp indicated by the gray box. Significance was tested against both controls in individual *t* tests indicated by the color of the text.

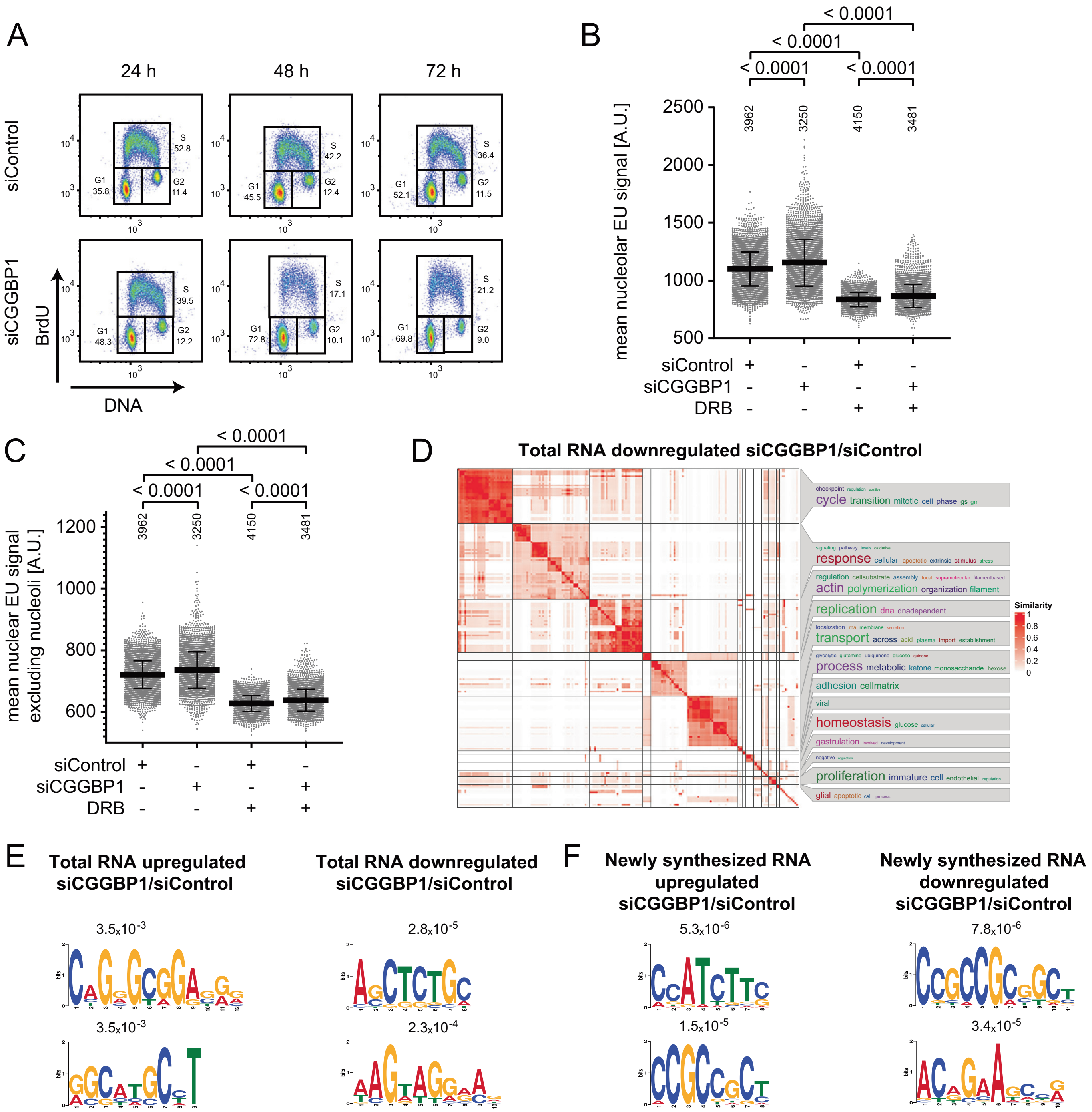

Figure EV2. CGGBP1 depletion leads to changes in transcriptional activity.

(**A**) BrdU cell cycle flow cytometry profile plots of U-2OS cells upon treatment with siControl or siCGGBP1 for 24, 48 and 72 h. The percentage of cells in G1, S or G2 are provided next to the gates. $N = 1$. (**B**) Quantification of mean nucleolar EU signal from Fig. 2C. For transcriptional inhibition, 100 μM DRB was added 2 h before fixation. Data is represented as mean ± standard deviation. Statistical significance was calculated using one-way ANOVA. Data is pooled from two technical replicates. Total number of analyzed nuclei is shown above each condition. $N = 1$. (**C**) Quantification of mean nuclear EU signal excluding nucleoli from Fig. 2C. For transcriptional inhibition, 100 μM DRB was added 2 h before fixation. Data is represented as mean ± standard deviation. Statistical significance was calculated using one-way ANOVA. Data is pooled from two technical replicates. Total number of analyzed nuclei is shown above each condition. $N = 1$. (**D**) GO-term enrichment cluster analysis of the significantly downregulated genes (total RNA) upon CGGBP1 knockdown. Illustrated in the heatmap are the biological process GO terms clustered based on their similarity. (**E**) Motif probability graph and DNA sequence logo of the top 2 DNA sequence motifs enriched in significantly upregulated or downregulated genes (total RNA) upon CGGBP1 knockdown. (**F**) Motif probability graph and DNA sequence logo of the top 2 DNA sequence motifs enriched in significantly upregulated or downregulated genes (newly synthesized RNA) upon CGGBP1 knockdown.

 © The Author(s)

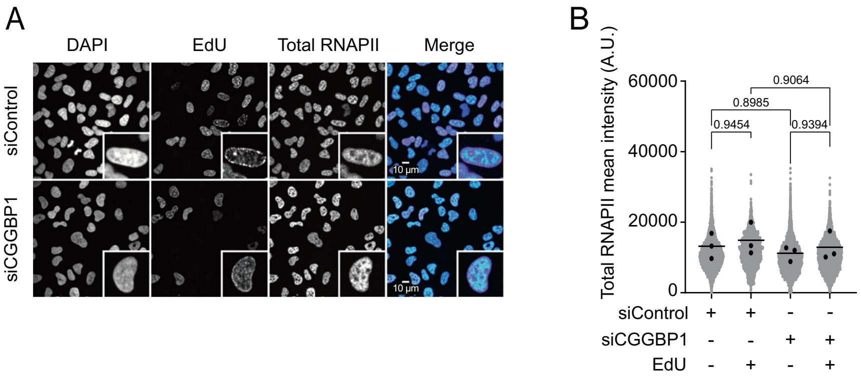

Figure EV3. Altering cellular CGGBP1 levels impacts the level of chromatin-bound RNAPII complexes.

(**A**) Example IF images of total RNAPII and EdU incorporation upon treatment of U-2OS cells with siControl or siCGGBP1 for 72 h. (**B**) Quantification of mean nuclear total RNAPII signal in EdU(−) and EdU(+) cells with siControl or siCGGBP1 from (**A**), $N = 3$, mean values per biological replicate depicted as dots. Bars indicate mean values with standard deviations (SD). Ordinary one-way ANOVA with Fisher's test.

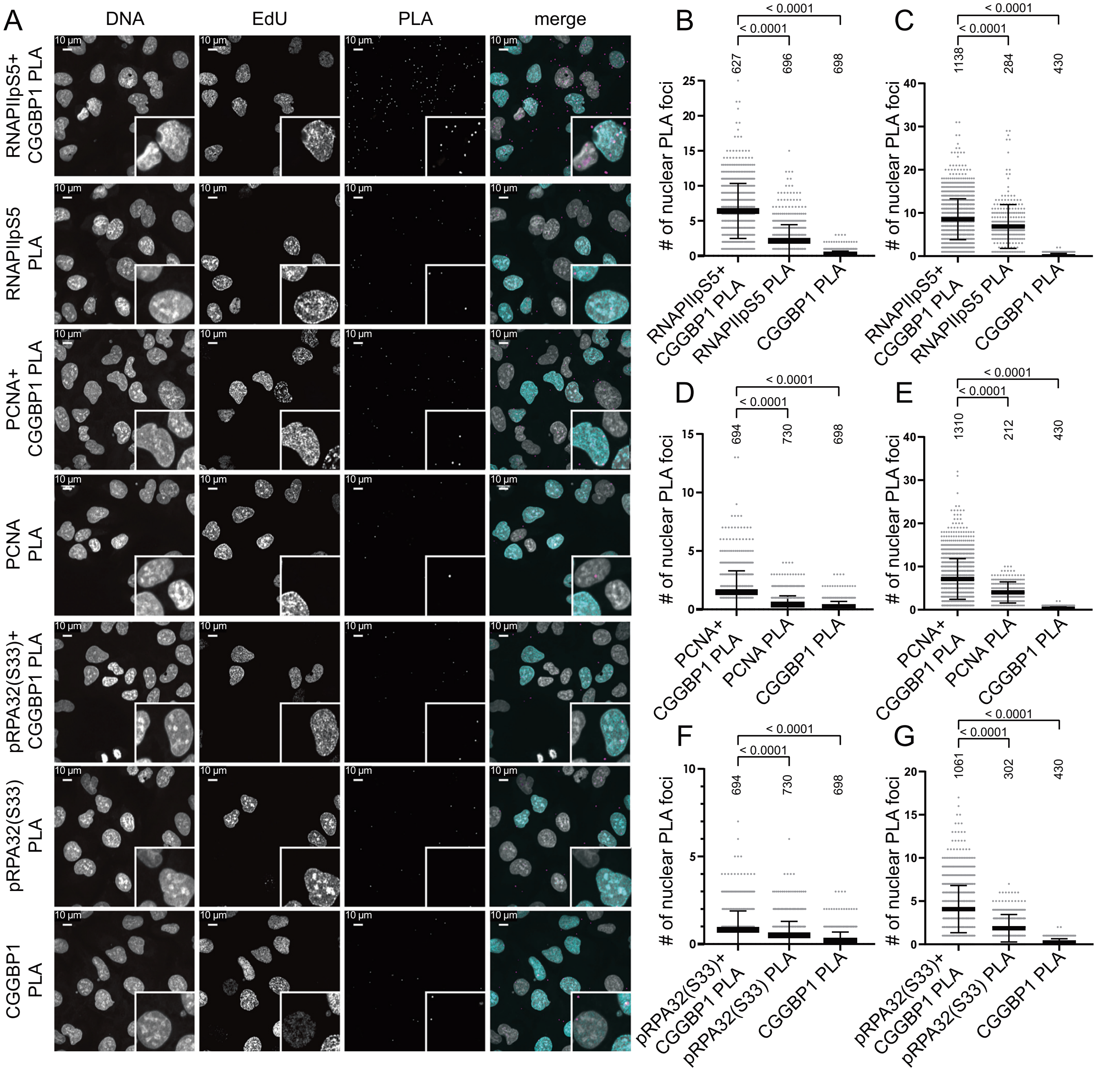

Figure EV4. CGGBP1 depletion leads to increased levels of transcription-replication interference.

(**A**) Example IF images of EdU incorporation and proximity ligation assay foci of the indicated antibody combination or single antibody controls in untreated U-2OS cells. (**B**) Quantification of RNAPII-pS5 – CGGBP1 PLA foci in all fields similar to (**A**). The single antibody controls are shown for both antibodies. Data is represented as mean ± standard deviation. Statistical significance was calculated using ordinary one-way ANOVA. (**C**) Biological replicate of (**B**). (**D**) Quantification of PCNA – CGGBP1 PLA foci in all fields similar to (**A**). The single antibody controls are shown for both antibodies. For comparison reasons, the same data of the single CGGBP1 antibody PLA control as in (**B**) is shown. Data is represented as mean ± standard deviation. Statistical significance was calculated using ordinary one-way ANOVA. (**E**) Biological replicate of (**D**) For comparison reasons, the same data of the single CGGBP1 antibody PLA control as in (**C**) is shown. (**F**) Quantification of pRPA32(S33) – CGGBP1 PLA foci in all fields similar to (**A**). The single antibody controls are shown for both antibodies. For comparison reasons, the same data of the single CGGBP1 antibody PLA control as in (**B**) is shown. Data is represented as mean ± standard deviation. Statistical significance was calculated using ordinary one-way ANOVA. (**G**) Biological replicate of (**F**). For comparison reasons, the same data of the single CGGBP1 antibody PLA control as in (**C**) is shown.

© The Author(s)

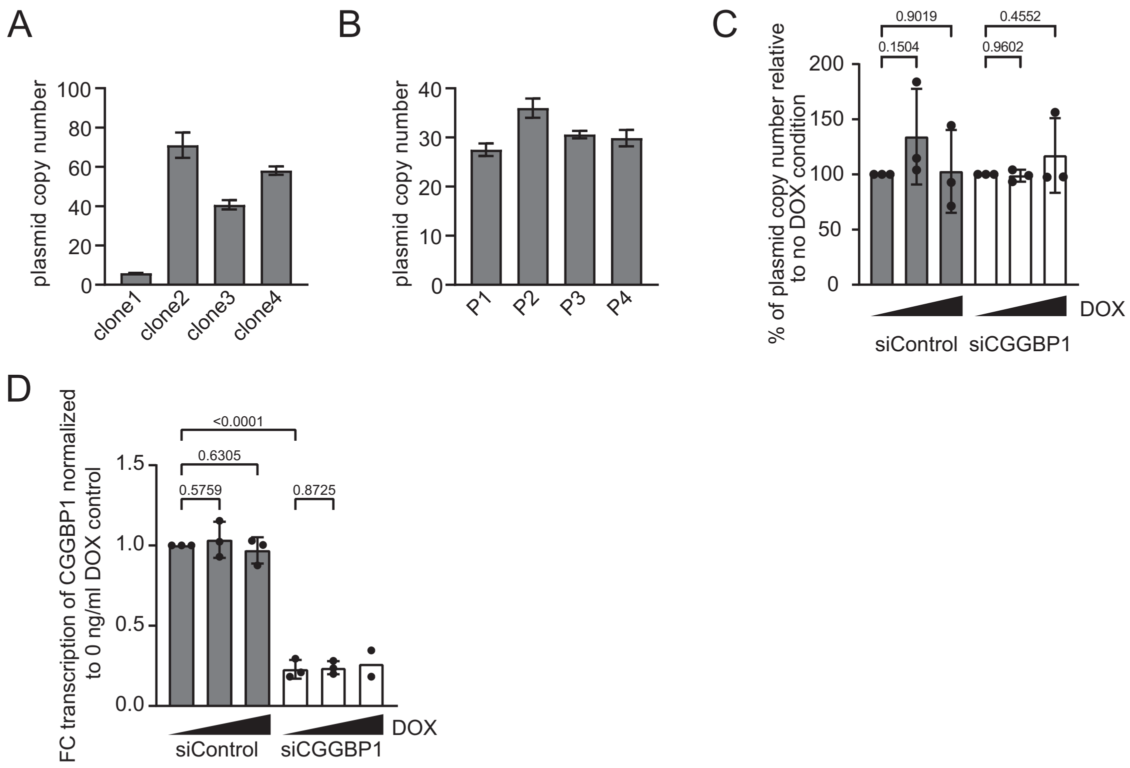

Figure EV5. CGGBP1 binding opposes secondary structure formation and promotes transcription elongation on an episomal system.

(**A**) Initial plasmid copy numbers of different U-2OS Tet-ON monoclonal cell lines carrying the episomal system measured by qPCR (see Fig. 5A). Plasmid copy numbers were calculated with the relative ratio 2× OriP/β-actin. Data is represented as mean ± standard deviation. $N = 3$. (**B**) Plasmid copy numbers of U-2OS Tet-ON pHU43 clone 2 during passaging measured by qPCR. The time between passages was 3-4 days. DOX and siRNA treatment experiments were done between passages 1 and 4. Data is represented as mean ± standard deviation. $N = 3$. (**C**) Plasmid copy number changes during DOX treatment of U-2OS Tet-ON pHU43 clone 2. Data is represented as mean ± standard deviation. $N = 3$. (**D**) Gene expression of CGGBP1 measured by RT-qPCR of cDNA from U-2OS Tet-ON pHU43 clone 2 carrying the episomal system and treated with siControl or siCGGBP1 for 72 h. Cells were treated with 0, 100 or 1000 ng/ml DOX for 72 h. Shown is the fold change relative to MCM3 mRNA levels and normalized to the 0 ng/ml DOX control. Data is represented as mean ± standard deviation. Statistical significance was calculated using one-way ANOVA. $N = 3$, except for the siCGGBP1 1000 ng/ml condition. See Fig. EV5A,B for characterization of U-2OS Tet-ON pHU43 clone 2.

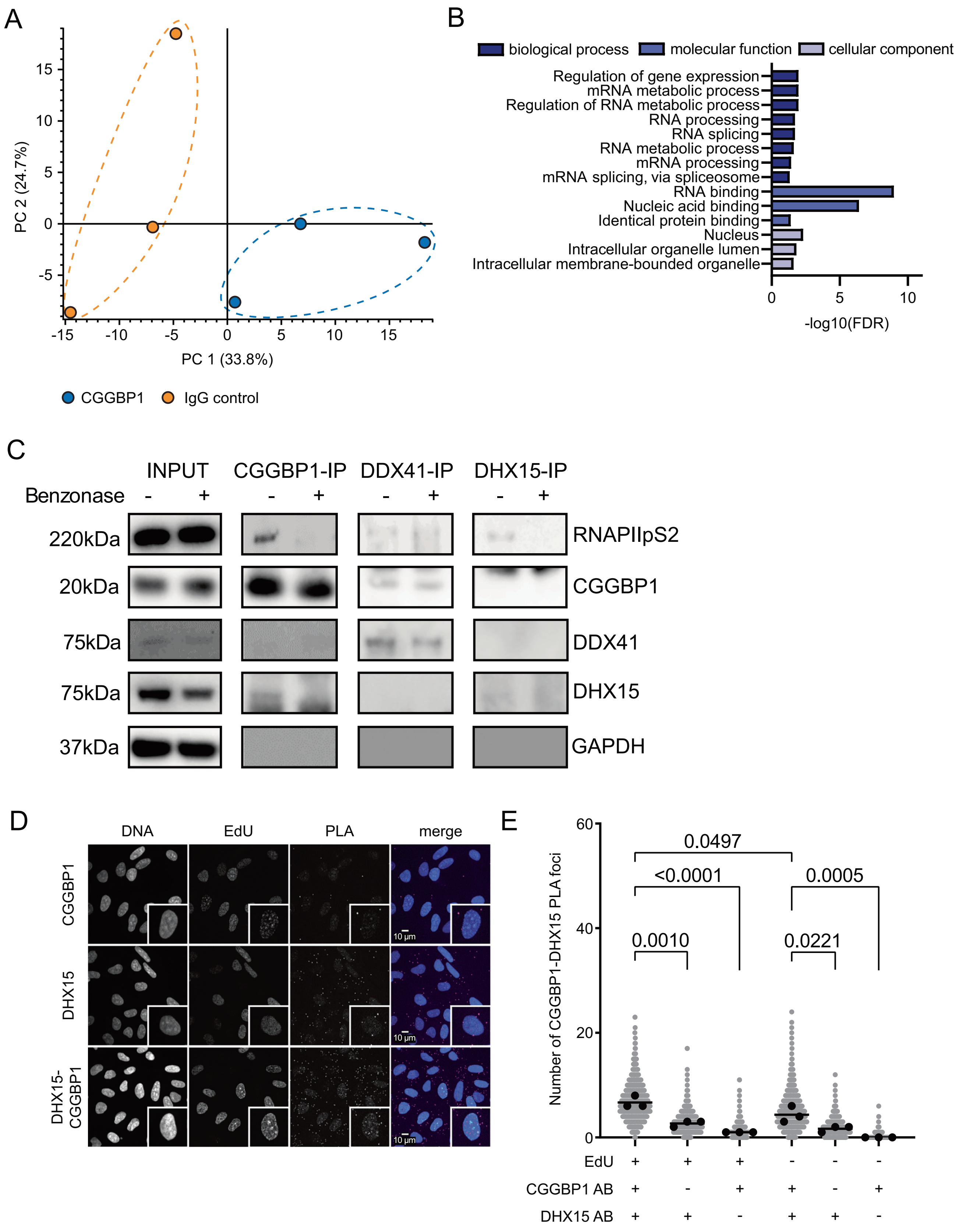
A
PC 2 (24.7%)
PC 1 (33.8%)
CGGBP1
IgG control
B
biological process
molecular function
cellular component
Regulation of gene expression
mRNA metabolic process
Regulation of RNA metabolic process
RNA processing
RNA splicing
RNA metabolic process
mRNA processing
mRNA splicing, via spliceosome
RNA binding
Nucleic acid binding
Identical protein binding
Nucleus
Intracellular organelle lumen
Intracellular membrane-bounded organelle
-log10(FDR)
C
INPUT
CGGBP1-IP
DDX41-IP
DHX15-IP
Benzonase
220kDa
20kDa
75kDa
75kDa
37kDa
RNAPIIpS2
CGGBP1
DDX41
DHX15
GAPDH
D
DNA
EdU
PLA
merge
CGGBP1
DHX15
DHX15-CGGBP1
10 µm
E
Number of CGGBP1-DHX15 PLA foci
0.0497
<0.0001
0.0005
0.0010
0.0221
EdU
CGGBP1 AB
DHX15 AB

 © The Author(s)

Figure EV6. CGGBP1 interactome is enriched for RNA:DNA helicase enzymes.

(**A**) Principal component analysis of CGGBP1-IP mass spectrometry replicates for siCGGBP1 and siControl samples. (**B**) GO-term enrichment analysis of the significantly enriched CGGBP1-interacting proteins. Illustrated are the false discovery rates for the top hits in the subcategories biological process, molecular function and cellular component. (**C**) Representative Western Blot of U-2OS wholce cell lysates and Co-IP samples with and without benzonase treatment showing RNAPII, CGGBP1, DDX41, DHX15 and GAPDH signals of Input, CGGBP1-IP, DDX41-IP and DHX15-IP samples. $N = 3$. (**D**) Example IF images of EdU incorporation and DHX15-CGGBP1 proximity ligation assay foci in U-2OS cells. (**E**) Quantification of nuclear DHX15-CGGBP1 PLA foci in EdU- and EdU+ cells from (**D**). Data is represented as mean of three biological replicates ± standard deviation. Statistical significance was calculated using one-way ANOVA.

© The Author(s)

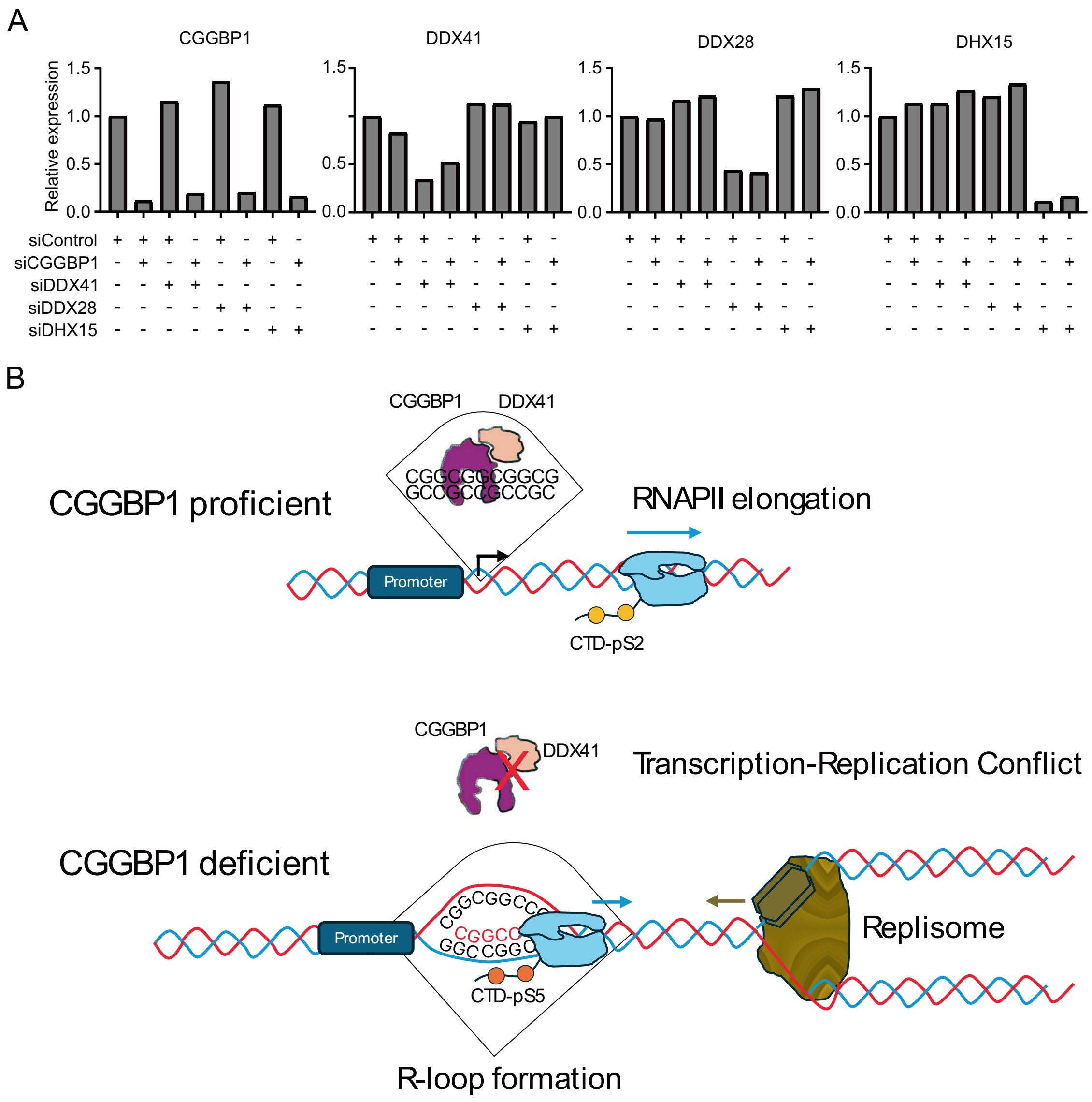

Figure EV7. DDX41 works in concert with CGGBP1 to counteract the formation of R-loops at CGG-repeat-containing RNAPII promoters.

(**A**) Gene expression of CGGBP1, DDX41, DDX28 and DHX15 measured by RT-qPCR of cDNA from U-2OS Tet-ON pHU43 clone 2 carrying the episomal system and treated with the indicated siRNA combinations for 72 h. Shown is the relative expression normalized to the siControl condition. (**B**) Working model of CGGBP1 counteracting the formation of R-loops and TRCs by recruitment of DDX41 RNA:DNA helicase at promoter CGG-repeat tracts. CGGBP1-proficient cells allow recruitment of DDX41 and thereby preventing the formation of R-loops and potential other DNA secondary structures at short CGG-repeat-containing promoters. CGGBP1-deficient cells cannot recruit DDX41 and therefore accumulate R-loops at short CGG-repeat-containing promoters. This leads to an accumulation of RNAPII at the promoters and increased interference with DNA replication upon entry into S-phase cells.

 © The Author(s)
